# DISCO-SCA and Properly Applied GSVD as Swinging Methods to Find Common and Distinctive Processes

**DOI:** 10.1371/journal.pone.0037840

**Published:** 2012-05-31

**Authors:** Katrijn Van Deun, Iven Van Mechelen, Lieven Thorrez, Martijn Schouteden, Bart De Moor, Mariët J. van der Werf, Lieven De Lathauwer, Age K. Smilde, Henk A. L. Kiers

**Affiliations:** 1 Department of Psychology, Katholieke Universiteit Leuven, Leuven, Belgium; 2 Translational Cardiomyology, Stem Cell Institute Leuven Department of Development and Regeneration, Katholieke Universiteit Leuven, Leuven, Belgium; 3 Department Electrical Engineering (ESAT-SCD), Katholieke Universiteit Leuven/IBBT Future Health Department, Kasteelpark Arenberg 10, Leuven, Belgium; 4 Interdisiciplinary Research Center, Katholieke Universiteit Leuven Campus Kortrijk, Kortrijk, Belgium; 5 TNO, Quality of Life, Zeist, The Netherlands; 6 Group Science, Engineering and Technology, Katholieke Universiteit Leuven Campus Kortrijk, Kortrijk, Belgium; 7 Biosystems data analysis, Swammerdam Institute for Life Sciences, University of Amsterdam, Amsterdam, The Netherlands; 8 Heymans Institute, University of Groningen, Groningen, The Netherlands; University of Rome, Italy

## Abstract

**Background:**

In systems biology it is common to obtain for the same set of biological entities information from multiple sources. Examples include expression data for the same set of orthologous genes screened in different organisms and data on the same set of culture samples obtained with different high-throughput techniques. A major challenge is to find the important biological processes underlying the data and to disentangle therein processes common to all data sources and processes distinctive for a specific source. Recently, two promising simultaneous data integration methods have been proposed to attain this goal, namely generalized singular value decomposition (GSVD) and simultaneous component analysis with rotation to common and distinctive components (DISCO-SCA).

**Results:**

Both theoretical analyses and applications to biologically relevant data show that: (1) straightforward applications of GSVD yield unsatisfactory results, (2) DISCO-SCA performs well, (3) provided proper pre-processing and algorithmic adaptations, GSVD reaches a performance level similar to that of DISCO-SCA, and (4) DISCO-SCA is directly generalizable to more than two data sources. The biological relevance of DISCO-SCA is illustrated with two applications. First, in a setting of comparative genomics, it is shown that DISCO-SCA recovers a common theme of cell cycle progression and a yeast-specific response to pheromones. The biological annotation was obtained by applying Gene Set Enrichment Analysis in an appropriate way. Second, in an application of DISCO-SCA to metabolomics data for *Escherichia coli* obtained with two different chemical analysis platforms, it is illustrated that the metabolites involved in some of the biological processes underlying the data are detected by one of the two platforms only; therefore, platforms for microbial metabolomics should be tailored to the biological question.

**Conclusions:**

Both DISCO-SCA and properly applied GSVD are promising integrative methods for finding common and distinctive processes in multisource data. Open source code for both methods is provided.

## Introduction

In biology several important research questions focus on the integration of data that come from different sources (e.g., organisms, measurement platforms) but that are gathered under the same set of conditions or for the same set of biomolecules (e.g., genes, metabolites). Examples where different organisms are compared include the study of orthologous genes [1]–[3] and the comparison of the genome wide expression of yeast and human for the same set of equivalent cell-cycle states [4]. Examples where different measurement platforms form the different sources are the integration of ChIP-chip, motif, and expression data collected for the same set of genes [5] and metabolomics data obtained for the same set of Escherichia coli samples using either gas chromatography mass spectrometry (GC-MS) or liquid chromatography mass spectrometry (LC-MS) as a chemical analysis method. In all these examples, the use of multiple sources to collect data on the same set of entities leads to data consisting of multiple data blocks; this introduces a problem of data fusion.

Important biological questions for such multisource data often aim at 1) finding the important biological processes underlying the data as a whole and 2) disentangling therein the biological processes shared between the different sources and the biological processes specific for a particular source. For example, the interspecies comparative analysis of the expression of orthologous genes aims at finding processes that are conserved (common) and processes that are diverged (distinctive for both organisms); see [6]. The comparison of the genomewide expression between yeast and human in equivalent cell-cycle states also aimed at common (e.g., cell-cycle oscillations) and distinctive (e.g., yeast-specific pheromone response) processes ([4]). In the example of GC-MS and LC-MS data sets, it may be of interest to find the biological processes of which the associated metabolites are targeted by only one of the analytical methods [7].

A fruitful method to tackle the problem of finding the important biological mechanisms that underlie a single data block is SVD (PCA) [8]. This method and variants thereof (e.g., nonnegative matrix factorization [9]) have also been used in the context of data integration by using two-step approaches. Either by first applying a separate SVD to each data block and subsequently comparing the results [10] or by first building a model based on one particular data block and then projecting the remaining data blocks on the model [11]. As these approaches do not rely on a common model structure that holds for all data blocks simultaneously, they are less well suited to find the processes underlying all data and disentangling therein processes shared between all data sources and processes distinctive for a particular source. In fact, only few methods have been proposed that do not require prior information and that model all data simultaneously, instead of using a segmented strategy. [12] proposed a method that uses a simultaneous strategy to find the common components but that models the distinctive components for each data block separately conditional upon the common components. Similarly, [13] proposed a method that uses a segmented strategy to extract the distinctive components of each data block separately, and subsequently, uses a simultaneous strategy to extract the common components. To our knowledge, only two methods have been proposed that fit a fully integrated simultaneous modelling framework and that disentangle common and distinctive sources of variation. First, it was proposed to use the generalized singular value decomposition (GSVD) as a generalization of SVD to two data blocks that simultaneously gives an answer to the second question of finding common and distinctive mechanisms [4]. Second, the idea to reveal common and distinctive processes by a proper rotation of the components resulting from a simultaneous components analysis (SCA) was introduced by [14] under the name DISCO-SCA. In this paper we will compare for the first time both approaches and clearly show that standard application of the GSVD may yield components that barely approximate the data and that do not capture the underlying processes. On the other hand, DISCO-SCA gives an optimal (in the least squares sense) approximation of the data and yields a good recovery of the underlying processes. As we will show, the GSVD can be adapted to a method closely related to DISCO-SCA [15].

In this paper, we first formally introduce the methods with emphasis on data analysis aspects. Then the GSVD and DISCO-SCA decompositions are formulated. The performance of both methods is compared on simulated data and the adapted GSVD is introduced and applied to the same simulated data. Two empirical applications, one on the genomewide expression of human and yeast for synchronized cell-cycle states [4], and one on the metabolome of *E. coli* measured by GC-MS and LC-MS [16] are discussed.

## Methods

We will rely on the following notation: matrices are denoted by bold uppercase characters, vectors by bold lower case characters, and scalars (single numbers) by italic characters [17]. The superscript ^T^ is used to denote the transpose of matrices and vectors. The cardinality of an index is denoted by the capital of the letter used to run the index. For example, the k^th^ data block is denoted by **X**_k_ with *k* running from 1 to *K*. The data of interest in this paper consist of two data matrices, **X**_1_ and **X**_2_, that have a common set of entities. Let this common set refer to the columns of the data matrices. Then, **X**_1_ is of size *I*_1_×*J* (i.e., **X**_1_ has *I*_1_ rows and *J* columns) and **X**_2_ is of size *I*_2_×*J* where in general *I*_1_≠*I*_2_.

### 1. Generalized singular value decomposition

A generalization of the singular value decomposition to a simultaneous decomposition of two data matrices with a common set of column entities is offered by the generalized singular value decomposition. The original GSVD as introduced by [18] requires that *I*_2_≥*J*; [19] generalized the GSVD to *I*_2_ of any size. Here, we follow their presentation of the GSVD.

#### Decomposition

Let *Q* be the rank of the concatenated matrix **X**_c_ = [**X**_1_^T^ **X**_2_^T^]^T^, then
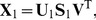
(1)

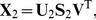
(2)with **U**_1_ (of size *I*_1_×*I*_1_) and **U**_2_ (of size *I*_2_×*I*_2_) orthogonal, **S**_1_ (*I*_1_×*Q*) and **S**_2_ (*I*_2_×*Q*) matrices with zeros everywhere except for the diagonal positions of the square matrix containing the first *Q* rows and columns of **S**_1_ and the last *Q* rows and columns of **S**_2_. For these diagonal positions, it holds that *s* ^2^_1qq_+*s* ^2^_2qq_ = 1 (see [19] for a proof). **V** (*J*×*Q*) is a matrix of full rank that represents the common structure shared between **X**_1_ and **X**_2_.

#### Estimation

Several algorithms have been proposed to compute the GSVD, for an overview and comparison we refer to [20]. The results described in this paper were obtained using a MATLAB implementation of the algorithm described in [19], see section 1 in Information S1; the code is available at the end of Information S1. Note that MATLAB has a built-in GSVD function but this does not make any assumptions on the ranks of the data and therefore may yield results different from those described in the literature.

#### Properties

The GSVD is a full decomposition of the data blocks **X**_1_ and **X**_2_; unlike SVD, it does not give an optimal (in the least squares sense) rank *R* approximation in presence of noise correlated with the data. This is explained in Information S1 (section 2). In line with the general ideas underlying SVD and PCA [4], suggested to base the rank *R* GSVD approximation on the *R* components that account for the maximal amount of variation in the concatenated data. As proven in Information S1, the VAF in the concatenated data by the *q* ^th^ component equals 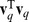, with **v**_q_ the *q* ^th^ vector of the common structure **V** in (4) and (5). It is important to realize that we are interested in approximating [**X**_1_^T^ **X**_2_^T^]^T^ and not, for example, in the derived data **X**_1_ **X**_2_^−1^ (assuming the special case of an invertible matrix **X**_2_). In the latter case, the GSVD gives the ordinary SVD of these derived data and thus can be used for the optimal approximation of **X**_1_ **X**_2_^−1^ (see [21] for an application).

Whether a component is common or distinctive, can be determined from the complementary values *s* ^2^_1qq_ and *s* ^2^_2qq_: If both *s* ^2^_1qq_ and *s* ^2^_2qq_ are close to 0.5, the component is common; if *s* ^2^_1qq_ is close to one (and thus *s* ^2^_2qq_ close to zero), the component is distinctive for **X**_1_; and if *s* ^2^_2qq_ is close to one, the component is distinctive for **X**_2_. In fact *s* ^2^_1qq_ (*s* ^2^_2qq_) can be interpreted as the proportion of VAF by GSVD component *q* in data block **X**_1_ (**X**_2_); see section 2.1. of Information S1.

### 2. Common and distinctive simultaneous components

Simultaneous component methods are a class of closely related integrative methods using a simultaneous modelling approach. Let the PCA decomposition of a single data block **X**_k_ into *R* components be
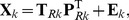
(3)with **T**_Rk_ of size *I*_k_×*R* and **P**_Rk_ of size *J×R*. All simultaneous component methods are a generalization of PCA to more than one data matrix by imposing **P**_R1_ = .. = **P**_RK_ = **P**_R_ on the matrix-specific PCA decompositions (3). We refer to [22] for a general framework and to [23] for the link between simultaneous component methods and Tucker-1 in case of three-way data.

#### Decomposition

Let *R* be the desired rank of the approximation of the data, then the simultaneous component decomposition of **X**_1_ and **X**_2_ is given by
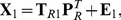
(4)

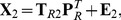
(5)with **P**_R_^T^ **P**_R_ = **I** in case the columns refer to the samples and [**T**_R1_^T^ **T**_R2_^T^][**T**_R1_^T^ **T**_R2_^T^]^T^ = **I** in case the columns refer to the variables. Note that the matrices **P**_R_ are the same for each data block which explicitly shows that the same low-dimensional space (of dimension *R*) is used for both data blocks with exactly the same component scores **P**_R_ (columns refer to the samples) or component loadings **P**_R_ (columns refer to the variables). The simultaneous components represent hidden biological processes underlying the data. The scores express how the samples are related to these simultaneous components, e.g., how strongly the underlying biological process is involved in the cellular state. The component loadings express how the variables are related to the simultaneous components, e.g., how strongly a gene is up- or down-regulated in the underlying biological process.

#### Estimation

The least squares estimation of the simultaneous component model with *R* components can be obtained by a SVD of the concatenated data **X**_c_ = **U**_c_ **S**_c_ **V**_c_^T^; in case the columns refer to the variables the concatenated matrix-specific component scores [**T**_R1_^T^ **T**_R2_^T^]^T^ are set equal to **U**_cR_ and the common loadings **P**_R_ (the same matrix of loadings is used for each data block) are set equal to **V**_cR_ **S**_cR_, in case they refer to the samples the loadings are [**T**_R1_^T^ **T**_R2_^T^]^T^ = **U**_cR_ **S**_cR_ and the component scores are **P**_R_ = **V**_cR_ (the same matrix of component scores is used for each data block).

#### Properties

The data are approximated by the *R* simultaneous components associated to the largest singular values of the concatenated data **X**_c_. This is the least squares approximation and thus yields the maximal variation accounted for in the concatenated data. The VAF by simultaneous component *r* equals *s* ^2^_crr_.

**Figure 1:**
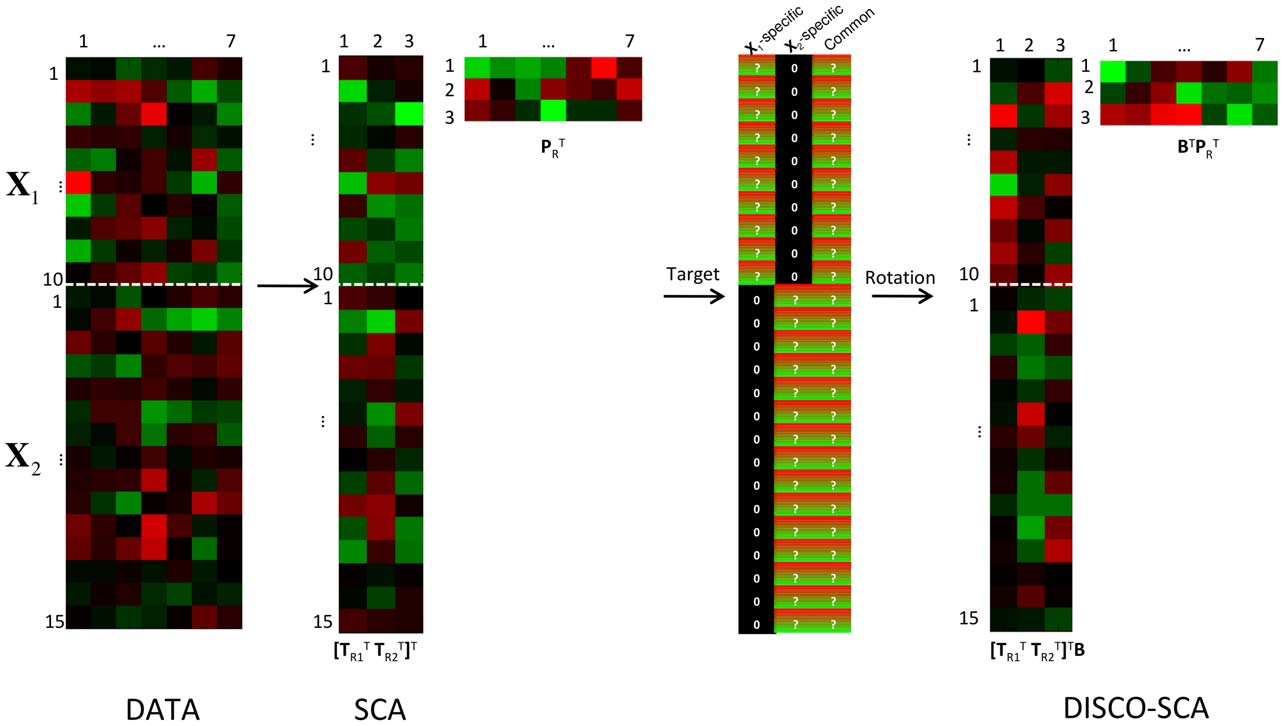
Visualization of the DISCO-SCA approach. Green is used for negative scores, black for (close to) zero scores, and red for positive scores. From left to right the method proceeds as follows: 1) The data are decomposed by SCA into matrices [**T**_R1_^T^ **T**_R2_^T^]^T^ and **P**_R_ of rank 3; 2) a partially specified target is defined with one specific component for each data block and one common component by specifying zeros for the parts in the specific components that correspond to the data block in which the component should not be present; 3) the matrix [**T**_R1_^T^ **T**_R2_^T^]^T^ is optimally rotated to the target resulting in the DISCO-SCA rotated matrix [**T**_R1_^T^ **T**_R2_^T^]^T^ **B** and the counter-rotated matrix **B** ^T^ **P**_R_^T^.

To find common and distinctive components, an additional step is introduced. Let **T**_R1_, **T**_R2_, and **P**_R_ give an optimal approximation of **X**_c_. Then [**T**_R1_^T^ **T**_R2_^T^]^T^ **B** and **P**_R_ **B** with **B** an (orthogonal) rotation matrix is optimal too. [14] propose to use this rotational freedom to rotate the simultaneous components to a partially specified target that defines distinctive components as components with all zero scores for the data block that they do not underlie (the remaining parts are left unspecified). Let **T**_Target_ denote the target, then the rotation matrix **B** is found using the following objective function [24]–[25],
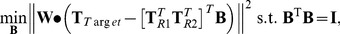
(6)with **W** a matrix with ones on the positions corresponding to the zeros specified in the target and with zeros on all remaining position and • denoting the elementwise product. In this way only those parts targeted to be zero add to the objective function; the remaining parts have no influence on it. An illustration of the decomposition of **X**_1_ and **X**_2_ in three simultaneous components and the associated target matrix for the case of one distinctive component for **X**_1_, one for **X**_2_, and one common component is given in Figure 1. For the estimation of **B**, we relied on an efficient numerical procedure that uses surrogate functions [26] (this is different from [14] which relies on general purpose rotation routines). When two or more components have the same status (e.g., two distinctive components for **X**_1_), this yields two equal columns in the target matrix and thus an indeterminacy due to the rotational invariance of the rotation criterion for these components. In such cases, we additionally rotate these components such that the first one explains maximal VAF in [**T**_R1_^T^ **T**_R2_^T^]^T^ and each subsequent component explains the maximal variation in the residual variation (confer singular value decomposition). The source code of the MATLAB implementation of this procedure is available in Information S1. If one knows how many common components and how many distinctive components for **X**_1_ and **X**_2_ underlie the data, the target and thus also the rotation matrix **B** follow automatically. When such information is not available, we propose a strategy based on a comparison of all possible targets. For each of the target rotations, the deviation of the rotated solution from the target is measured and the solution with the lowest deviation is retained. As a measure of deviation, we propose to take the maximum of the componentwise deviations. The componentwise deviation is measured 1) for a distinctive component, as the proportion of variation accounted for in the data block where the component should be absent and 2) for a common component, as the absolute difference in proportion of variation accounted for between the two data blocks (see sections 3.1 and 3.2. for an illustration of the strategy). In this way, the solution is retained that attains the target best for *each* component.

#### Related method

Our method is based on revealing underlying dimensions just as in factor analysis, with multigroup common factor analysis being its extension to the case of multiblock data. The main difference is that the dimensions in PCA are based on a linear combination of the variables and, more importantly, that our method can be applied directly while multigroup factor analysis is a supervised method that needs prior information (e.g., derived from PCA/SVD). Another difference is that multigroup factor analysis does not allow for factors that underlie all data blocks and that are at the same time outspokenly distinctive.

## Results

The performance of GSVD and DISCO-SCA is compared first for simulated data and then for two empirical data sets; a first empirical data set is on comparative genomics using synchronized cell cycle experiments for the human, and yeast genomes and a second one is on coupled metabolomics data as obtained for the same samples of *E. coli* but using different chemical analysis methods.

### 1. Simulated data example

The simulated data were generated under a model that fits both the GSVD and DISCO-SCA decompositions:
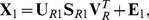
(7)

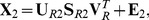
(8)with **U**_1_^T^ **U**_1_ = **I**, **U**_2_^T^ **U**_2_ = **I**, **V** ^T^ **V** = **I**, and with **X**_1_ of size 144×28 and **X**_2_ of size 44×28 and *R* = 6. **S**_1_ and **S**_2_ are diagonal matrices defining whether the components are common (equal singular values: *s*_1rr_ = *s*_2rr_) or distinctive (e.g., to impose a component that is distinctive for **X**_1_, *s*_1rr_ is set equal to a substantial value and *s*_2rr_ to zero). For example, in case of two distinctive components for **X**_1_, two distinctive for **X**_2_, and two common components:
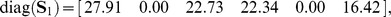


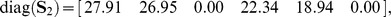
with the diag notation indicating that the diagonal of the matrix is taken. In order to generate more realistic data, we used the GC-MS and LC-MS data (to be discussed in the next section) in the following way: **U**_1_ and **U**_2_ are formed by the six left singular vectors associated to the six largest singular values of the GC-MS and LC-MS data respectively and **V** by the six right singular vectors associated to the largest singular values of the concatenated data. **E**_1_ and **E**_2_ were generated from a normal distribution with mean zero and variance such that these residual matrices account for 20 percent of the variation of **X**_1_ and **X**_2_ respectively. We generated a single representative datapair (**X**_1_,**X**_2_) for three different cases: only common components (*s*_1rr_ = *s*_2rr_ for all *i* = 1…6), only distinctive components (three for each data block), and a mix of common and distinctive components (two distinctive for each data block and two common). These three cases were analyzed both by the GSVD and DISCO-SCA.

A first measure of performance that we will consider, is the proportion of VAF because this indicates how well the data are approximated by the components. Table 1 summarizes the results: The three panels refer to the three simulated cases, namely only distinctive components in the top panel, a mix of common and distinctive components in the middle panel, and only common components in the bottom panel. Within each panel of Table 1, the proportion of variation that is accounted for in each datablock by each of the six components (the lines C1–C6) and their total (the ‘Total’ line) is displayed. In the first two columns this is for the imposed structure as represented by (7) and (8), in the middle two columns this is for the components as obtained by the GSVD, and in the last two columns this is for the components obtained from DISCO-SCA. Note that we selected both for the GSVD and DISCO-SCA the six components with the highest VAF in the concatenated data. We consider six components because this is the number of (common and distinctive) components we generated. Clearly DISCO-SCA outperforms GSVD when common components are present (middle and bottom panel): whereas the DISCO-SCA model fits well to the data in terms of the proportion of VAF, this is not the case for the GSVD. When all components are distinctive, both methods have a good fit.

**Table 1:** Proportion of variation accounted for by GSVD and DISCO-SCA for data generated to have a certain common and/or distinctive structure.

		Imposed		GSVD		DISCO-SCA		Ad. GSVD	
		X1	X2	X1	X2	X1	X2	X1	X2
**Distinctive**	**C1**	0.00	0.48	0.00	0.50	0.01	0.50	0.01	0.50
	**C2**	0.44	0.00	0.43	0.00	0.45	0.00	0.43	0.00
	**C3**	0.23	0.00	0.23	0.00	0.24	0.01	0.25	0.01
	**C4**	0.00	0.21	0.01	0.20	0.01	0.21	0.01	0.20
	**C5**	0.13	0.00	0.14	0.00	0.13	0.01	0.14	0.01
	**C6**	0.00	0.10	0.01	0.14	0.01	0.13	0.01	0.14
	**Total**	0.80	0.80	0.82	0.84	0.85	0.86	0.85	0.86
**Common & Distinctive**	**C1**	0.31	0.30	0.00	0.25	0.31	0.29	0.29	027
	**C2**	0.20	0.19	0.15	0.00	0.20	0.19	0.22	0.20
	**C3**	0.20	0.00	0.15	0.00	0.20	0.01	0.01	0.25
	**C4**	0.00	0.20	0.06	0.08	0.01	0.25	0.20	0.01
	**C5**	0.11	0.00	0.04	0.10	0.12	0.01	0.01	0.12
	**C6**	0.00	0.10	0.01	0.12	0.01	0.12	0.12	0.01
	**Total**	0.82	0.80	0.41	0.55	0.84	0.86	0.84	0.86
**Common**	**C1**	0.27	0.27	0.06	0.11	0.28	0.29	0.23	0.23
	**C2**	0.18	0.19	0.03	0.09	0.19	0.20	0.17	0.21
	**C3**	0.12	0.12	0.05	0.07	0.12	0.13	0.12	0.12
	**C4**	0.10	0.10	0.02	0.08	0.10	0.09	0.12	0.11
	**C5**	0.07	0.07	0.05	0.06	0.08	0.09	0.09	0.12
	**C6**	0.06	0.06	0.03	0.07	0.07	0.06	0.11	0.08
	**Total**	0.79	079	0.24	0.48	0.84	0.86	0.84	0.86

Proportion of variation accounted for (VAF) by each of six components (the lines C1–C6) and their total (the ‘Total’ line). These are the six components with highest VAF in the concatenated data. In the left panel we show the proportion of VAF as imposed on each data block, while in the three panels at the right we show the proportion of VAF as recovered by GSVD, DISCO-SCA, and adapted GSVD. In the top panel all imposed components are distinctive, in the middle panel the components are a mix of common and distinctive components, and in the bottom panel all components are common.

Second, the recovery of the underlying true structures (common scores **V**_R_ and block-specific scores **U**_R1_ and **U**_R2_) is evaluated. Block-specific parts for which s_krr_ **u**_rk_ is a zero vector, are not included in the calculations because **u**_rk_ is arbitrary in these cases. A suitable measure of performance, is the average Tucker's coefficient of congruence *φ* calculated between the columns of the true and recovered structures: it can be interpreted as a correlation (−1≤*φ*≤1) and is sensitive to rotations but not to scaling [27]. In the calculation of *φ*, we accounted for permutations and reflections of the components. The insensitivity to scaling makes that the recovery of **U**_R1_ and **U**_R2_ or of **U**_R1_ **S**_R1_ and **U**_R2_ **S**_R2_ yields the same value (unless one of the diagonal values of **S**_R1_ or **S**_R2_ equals zero). Table 2 reports the average *φ* value over 100 replications: The previously described data generation procedure was used but, in order to introduce variability over the replicate values in **U**_R1_, **U**_R2_, and **V**, these structures were derived from data based on sampling observations (with replacement) from the empirical GC-MS and LC-MS data (and hence not derived from the actual data as we did to produce the results in Table 1). Clearly, the recovery by GSVD in presence of common components is inferior to the recovery by DISCO-SCA and this is the more so the more common components are present.

**Table 2:** Recovery by GSVD and DISCO-SCA for data generated to have a certain common and/or distinctive structure.

	GSVD			DISCO-SCA		
	V_R_	U_R1_	U_R2_	V_R_	U_R1_	U_R2_
**Distinctive**	0.92	0.86	0.96	0.99	0.98	0.98
**Comm. & dist.**	0.82	0.56	0.67	0.99	0.98	0.98
**Common**	0.55	0.34	0.43	0.96	0.93	0.94

Congruence between constructed and recovered scores in each of the three conditions (only distinctive components, common and distinctive components, only common components).

Summarized, DISCO-SCA outperforms GSVD for the three simulated data cases as it gives a better approximation of the data and a better recovery of the underlying common and distinctive processes. The performance of the GSVD seems to deteriorate gradually in presence of common components. In section 2 of Information S1 we explain this trend of decreasing performance for more common components, in section 3 we show that the full (i.e., no rank reduction) GSVD decompositions are not unique in several cases, including the case of perfect common and perfect distinctive data and in section 4 we confirm the results using more extensive simulations.

Interestingly, as shown in section 5 of Information S1, the GSVD can be used in a way that it becomes a least-squares method, either by a minor modification of the algorithm of [19] (MATLAB code for this adapted algorithm is included at the end of Information S1), or equivalently by replacing the data by data derived from the rank *R* least squares approximation of the concatenated data, as suggested by [15], and followed by a GSVD algorithm that determines the rank of the data (e.g., the algorithm in [19]). Note that the first step in the latter approach is a simultaneous component analysis. Application of this adapted GSVD to the simulated data yields the proportion of VAF in the last columns of Table 1. As expected, the total VAF by the adapted GSVD is the same as for DISCO-SCA; both methods give an optimal approximation of the data. The recovery of the underlying structures by the adapted GSVD improves the GSVD though it remains below the recovery obtained with DISCO-SCA (averaged over 100 replications and over **V**_R_, **U**_R1_, and **U**_R2_, 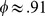 for the fully distinctive case, 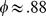 for the mixed case, and 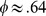 for the common case). This can be explained by the correspondence between the data generation mechanism and 1) the rotation to a target partially specified by zeros and 2) the additional rotation of DISCO-SCA for components of the same type to maximal VAF.

### 2. Empirical data examples

#### 2.1. Cross-species comparative genomics

A domain where it is of particular interest to find common and specific processes, is comparative genomics that studies the similarities and differences in the genomes of different species. A better understanding of the genome is gained by finding both evolutionary conserved and diverged elements. Also, knowledge of gene function in one species can be of use for the annotation of the gene in other species. Here, we will re-analyze data on the genome wide expression of the human and yeast genomes in cell-cycle experiments [4]. These involve the expression of 12 056 human and 4 523 yeast genes on 18 arrays (see [4] for more information and links to the data). For yeast, the culture was synchronized in the M/G1 phase and monitored over two cell cycle periods (7–119 minutes with a seven minutes interval); for human, cultures were synchronized initially in S phase and monitored over 34 hours with a 2 hour interval. As in [4] (see the supplementary Mathematica code of [4]), the first measurement of yeast is aligned with the first in human, the second with the second, and so on. We consider the first 12 time points yielding data of size 12 056×12 and 4523×12.

The data were mean-centered and scaled to sum-of-squares one per gene prior to the analysis. This avoids obtaining a dominant component that merely reflects absolute differences between genes and is the recommended preprocessing strategy for principal component analysis [28]. An additional interpretational advantage is that the loadings then equal the correlation of the gene expression profile with the components and thus allows for the use of enrichment tools that include the correlation as a metric to rank the genes. A further pre-processing step is that we divided each data block by the square root of the number of its genes to avoid that the GSVD and DISCO-SCA solutions are dominated by the much larger human data block [22]. Within each data block, the loadings then equal the correlations divided by the square root of the number of genes in the data block. Because this is a constant scaling factor within each data block and the enrichment analyses are conducted per block, this means that the correlation can still be used as a metric to rank the genes. Data obtained in this manner were used as input for the GSVD and DISCO-SCA analysis.

**Figure 2:**
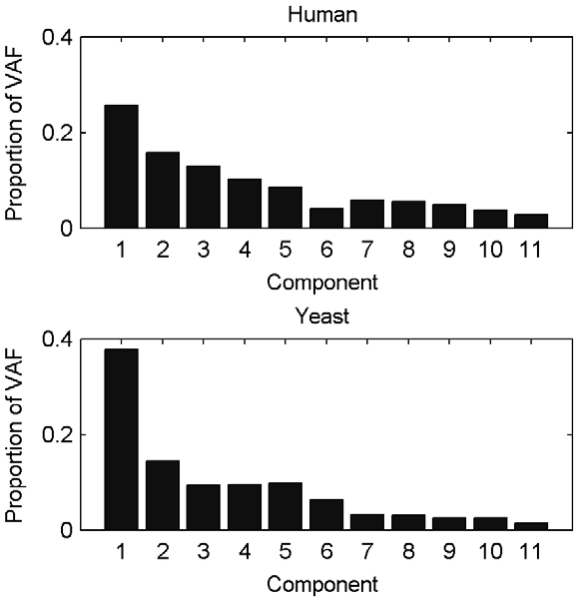
Proportion of variation accounted for by SCA in each data block. Upper panel : for the human data; lower panel: for the yeast data. The components are ordered according to the VAF for the concatenated data.

**Figure 3:**
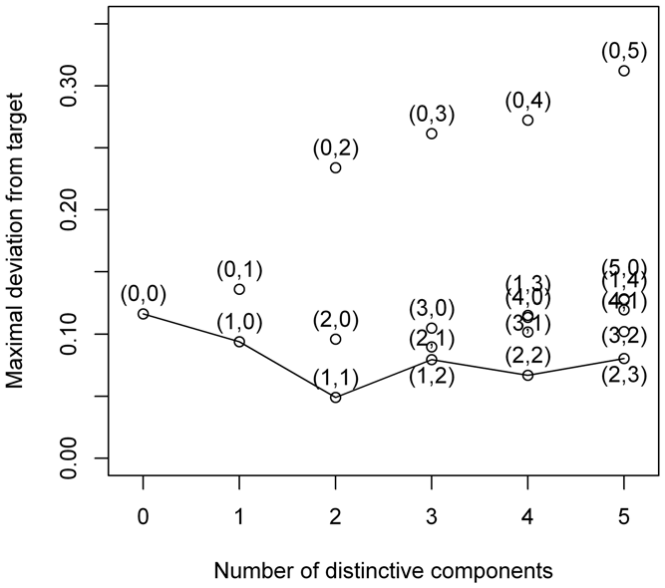
Deviation from the target structure in function of the number of distinctive components. All possible combinations of common and yeast and human-specific components in a model with five components are tried as a target for the DISCO rotation. The deviation of the DISCO-SCA solution from this target is displayed against the number of distinctive components. The targets are labelled by the numbers between brackets (the first number indicates the number of distinctive components for yeast, the second for human). The line connects solutions with the lowest deviation for a fixed number of distinctive components.

As a first step in the analyses, a decision has to be made on how many components underlie the data. Therefore, we subjected the data to a simultaneous component analysis and we inspected the VAF by each component in each data block to decide how many components explain the structural part in the data, see Figure 2 (with the components ordered in function of the overall VAF). This type of plot generalizes the scree graph used in principal component analysis (see [29]) for one data block to the case of multiple data blocks. In Figure 2, there is a more pronounced decrease in VAF after the first and fifth component for both the human and yeast data suggesting that there is a dominant process underlying the data and that the first five components are distinct from noise. Therefore, we will reduce the data to 5 components. Second, both for DISCO-SCA and the (adapted) GSVD, we have to decide how many of these five components are common, how many are specific for human and how many for yeast. For DISCO-SCA, we rotated the five simultaneous components to all 21 possible combinations of common, human specific, and yeast specific components. Figure 3 displays the deviation from the target against the number of distinctive components, defined as the deviation of the component with maximal deviation (for a distinctive component the deviation is measured as the proportion of VAF in the data block where the component should be absent while for a common component the deviation is measured as the difference in the proportion of VAF between the data blocks). The lowest value is obtained for the solution with three common components, one yeast-specific and one human-specific component. The VAF of this solution resembles the solution found by the adapted GSVD; see Table 3 that displays the VAF by DISCO-SCA, GSVD and adapted GSVD. Except for the GSVD, all solutions account for a large portion of the total variation (73 percent for the human data, 80 percent for the yeast data). Note that the human specific DISCO-SCA and adapted GSVD component does not have a clear status as it still accounts for a substantive amount of variation in the yeast data.

**Table 3:** Proportion of variance accounted for the comparative genomics data by DISCO-SCA, by the GSVD, and by the adapted GSVD.

	DISCO-SCA			GSVD			adapted GSVD		
	human	yeast	TOTAL	human	yeast	TOTAL	human	yeast	TOTAL
C1	0.23	0.23	0.23	0.08	0.10	0.10	0.11	0.10	0.10
C2	0.04	0.30	0.17	0.04	0.43	0.22	0.05	0.43	0.23
C3	0.22	0.08	0.15	0.22	0.03	0.14	0.35	0.08	0.22
C4	0.13	0.11	0.12	0.10	0.08	0.08	0.13	0.09	0.11
C5	0.10	0.10	0.10	0.09	0.09	0.09	0.09	0.10	0.09
**Total**	**0.73**	**0.81**	**0.77**	**0.52**	**0.72**	**0.63**	**0.73**	**0.81**	**0.76**

Proportion of variance accounted for the comparative genomics data by DISCO-SCA (with 3 common components: C1, C4, C5; one human component: C3; and one yeast component: C2), and by the GSVD and adapted GSVD. The components are ordered to have maximum congruence between the different analysis methods.

**Figure 4:**
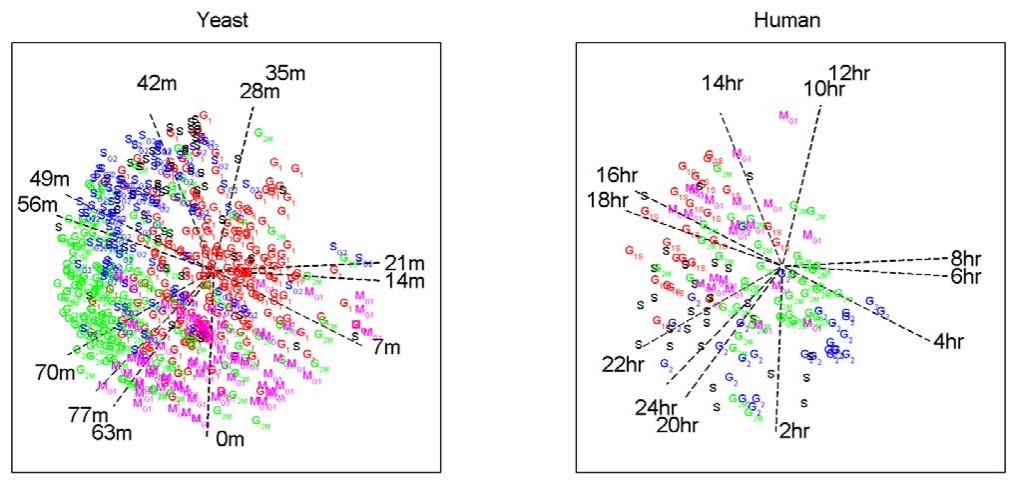
Common cell cycle space as found with DISCO-SCA. Both the time points and the cell cycle annotated genes are displayed with respect to the first two common components. The time points are displayed as oriented vectors; the genes as labelled points. The gene labels refer to phases of the cell cycle: M (magenta), M/G1 (magenta), G1 (red), G1/S (red), S (black), S/G2 (blue), G2 (blue), G2/M (green).

**Figure 5:**
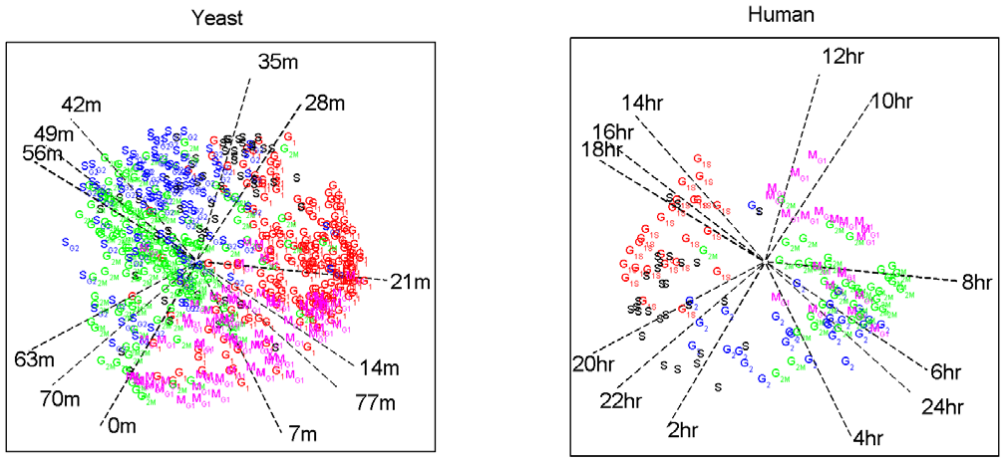
Common cell cycle space as found with the adapted GSVD. Both the time points and the cell cycle annotated genes are displayed with respect to the first two common components. The time points are displayed as oriented vectors; the genes as labelled points. The gene labels refer to phases of the cell cycle: M (magenta), M/G1 (magenta), G1 (red), G1/S (red), S (black), S/G2 (blue), G2 (blue), G2/M (green).

**Figure 6:**
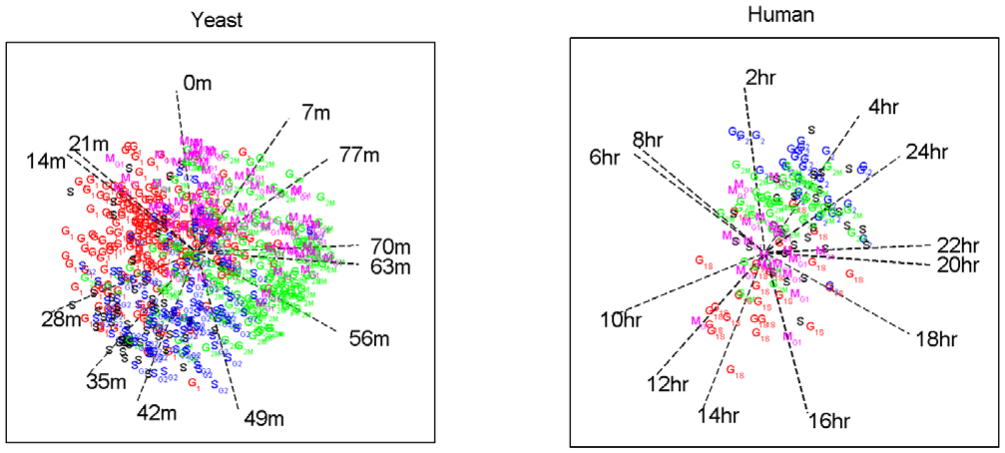
Common cell cycle space as found with the naïve GSVD. Both the time points and the cell cycle annotated genes are displayed with respect to the first two common components. The time points are displayed as oriented vectors; the genes as labelled points. The gene labels refer to phases of the cell cycle: M (magenta), M/G1 (magenta), G1 (red), G1/S (red), S (black), S/G2 (blue), G2 (blue), G2/M (green).

The biological validation and the annotation of the components is based on a gene set enrichment analysis using the correlation of the expression profile with the component scores as a metric for ranking the genes. As mentioned above, with proper pre-processing of the data, the correlation coincides with the loading of the gene on the component: higher loadings (in absolute value) indicating that the gene is more important for the component. This makes existing tools for enrichment analysis that include the correlation as a metric (for example GSEA [30], [31]) proper tools for the analysis of PCA based methods. GSEA also includes the option to use a self-defined ranking of the genes; this was used for the annotation of the naive and adapted GSVD results. The common components are significantly enriched (FWER<.05; this statistic is based on a permutation test where the ranks for the biological gene sets are compared to ranks for randomly created gene sets) in gene ontology terms related to the cell cycle (e.g. Cell cycle, DNA replication, Mitosis, M phase; see Table S1 and Table S2). GSVD finds fewer terms than DISCO-SCA and the adapted GSVD. All methods are able to identify the relation of ribosome biogenesis to the yeast cell cycle [32] but most terms related to ribosomal RNA are identified by DISCO-SCA. Only DISCO-SCA retrieves the yeast-specific response to pheromones, which is consistent with [4]. Further support of a common cell cycle space is given by Figures 4, 5, and 6 that plot the first two common components found by the DISCO-SCA (Figure 4), adapted GSVD (Figure 5), and the GSVD (Figure 6). Each panel depicts the time points as oriented vectors defined by the component scores of the (first) two common components and the genes - annotated for their cell cycle related function - as the loadings on these components. Panels at the left refer to the yeast data, at the right to the human data. The simultaneous modelling approach is reflected in the positions of the time points: these are exactly the same for both species (although differentially labelled). In general the ordering of the time points is that of a clock where the latest time folds back to the earliest time. Similar gene labels cluster together and these clusters are ordered according to the cell cycle phase. To assess the cluster-quality of the labelled genes in Figures 4, 5, and 6, we checked whether genes with the same cell-cycle phase label appear close together and well-separated from genes with another label. As a measure, we used the correlation between, on the one hand, closeness between the pairs of labelled genes in the modelled space with, and, on the other hand, a binary vector indicating whether the pair has the same (0) or different (1) cell cycle phase annotation [33]. As a measure of closeness between a pair of labelled genes, we calculated the cross product of the loadings in the five-dimensional solution space. This correlation amounts for the human data to .45 for DISCO-SCA and the adapted GSVD and to .39 for the GSVD; for the yeast data it amounts to .40 for DISCO-SCA, .38 for the adapted GSVD, and .32 for the GSVD.

In this particular example with only 12 time points, the performance of the GSVD is not much worse than that of the adapted GSVD or DISCO-SCA. As illustrated in Information S1 (see section 4.1.), the performance deteriorates with an increasing number of data columns. While DISCO-SCA and the adapted GSVD still recover the common cell cycle space when including all 18 time points in the analysis, this is no longer the case for the GSVD. As previously, we assessed the quality of clustering of the cell cycle phase annotations with the correlation of the cross products calculated between pairs of genes in the five component space with a binary vector indicating whether the pair of genes has the same (0) or different (1) annotation. This correlation drops, for the human data, drastically to 0.08 for the GSVD and just a little (to 0.42) for DISCO-SCA and the adapted GSVD; for the yeast data it drops to 0.20 for the GSVD and to 0.35 for DISCO-SCA and the adapted GSVD.

#### 2.2. Metabolomics data

The metabolome composition of 28 samples of *E. coli*, collected under different environmental conditions and harvested at different elapsed fermentation times was analyzed using mass spectrometry (MS) in combination with on the one hand gas chromatography (GC) and on the other hand liquid chromatography (LC) as a separation method [16]. This resulted in two coupled data blocks: a GC-MS block with the peak areas of 144 metabolites in the 28 conditions and a LC-MS block with the peak areas of 44 metabolites in these same conditions. The GC-MS and LC-MS methods used in this study were complementary methods and detected in general different classes of metabolites although some classes are detected by both methods [7]. Full metabolome coverage by applying as many chemical analysis methods as possible is in practice generally too expensive; therefore it is useful to know which platform targets the important metabolites in a specific biological setting [7]. Here we will illustrate the use of DISCO-SCA to find the biological processes targeted by GC-MS, by LC-MS, and by both detection methods. In the data considered here, only those metabolites that were detected in at least 20 percent of the experiments were used; furthermore, the data were manually curated and normalized [34]. Measurement values below the detection threshold were set equal to one half of the smallest detected value [16]. To deal with skewness and asymmetry, the square root of each value was taken [27]. Because the metabolites differ largely in abundance and we do not want the most abundant metabolites to dominate the analysis, the values were mean centered and scaled to sum of squares one per metabolite. Similarly, because the block of GC data is much larger, each block was scaled to sum of squares one.

**Figure 7:**
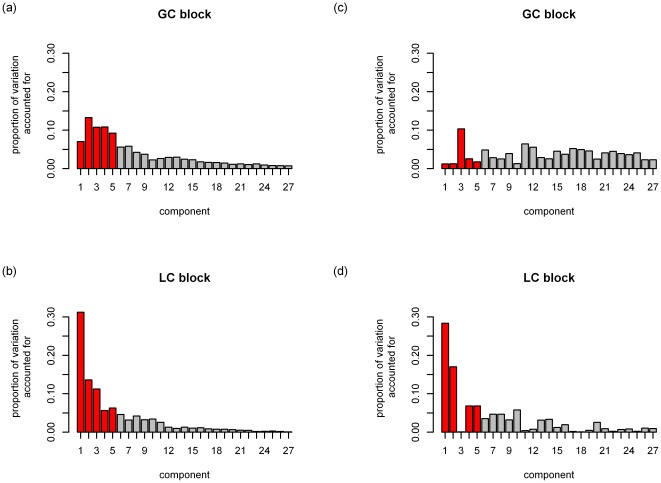
Proportion of variation accounted for in each data block. Panel (a): by SCA for the GC data; panel (b): by SCA for the LC data; panel (c): by GSVD for the GC data; panel (d): by GSVD for the LC data. For each method, the components are ordered according to the VAF in the concatenated data. The bars for the rank 5 approximation are coloured in red.

A first question that has to be answered is how many dimensions are needed to describe the important processes that underlie the fused GC-MS/LC-MS data. In the literature on simultaneous component analysis few guidelines are offered to assist in this choice. Here, we make use of a generalization of the scree graph used in principal component analysis (see [29] for a discussion on selecting the number of principal components) to simultaneous component analysis by displaying the proportion of VAF by the simultaneous components in each of the data blocks (see [22]): Figure 7 displays for each component the proportion of VAF in the GC data (upper panels) and the LC data (lower panels). Panels at the left refer to the simultaneous components while panels at the right refer to the GSVD components. Both for SCA and GSVD, the components are ordered according to the proportion of VAF in the concatenated data. For SCA (the left panels (a) and (b) in Figure 7) it seems that there is a sudden decrease after the fifth component for GC and after the third for LC. As we are also interested in components that are distinctive for GC, we will retain five components in the approximation. In total these five components account for 51% (GC) and 68% (LC) of the variation (note that the adapted GSVD for *R* = 5 would give the same total VAF). For the GSVD, the five components that account for maximal variation in the concatenated data, account for 17% (GC) and 59% (LC) of the variation respectively. This confirms what we observed for the simulated data: DISCO-SCA (and thus also the adapted GSVD) gives a better approximation of the data; the difference in VAF for the GC data is large.

**Figure 8:**
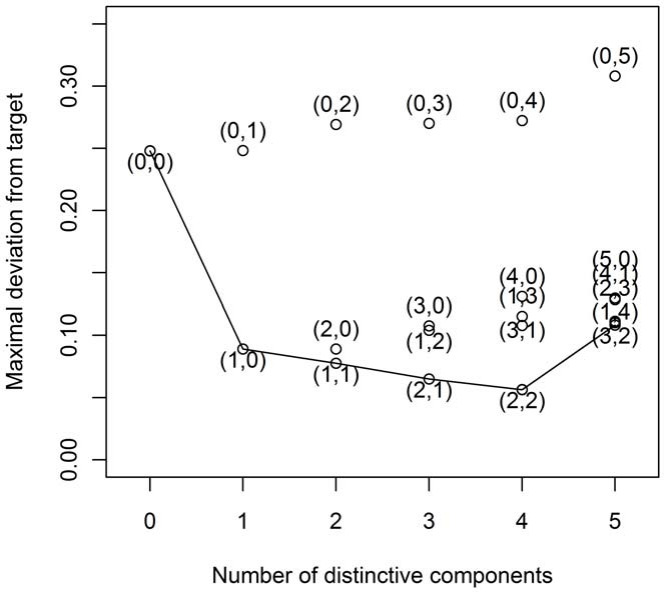
Deviation from the target structure in function of the number of distinctive components. All possible combinations of common and yeast and human-specific components in a model with five components are tried as a target for the DISCO rotation. The deviation of the DISCO-SCA solution from this target is displayed against the number of distinctive components. The targets are labelled by the numbers between brackets (the first number indicates the number of distinctive components for GC, the second for LC). The line connects solutions with the lowest deviation for a fixed number of distinctive components.

The simultaneous components are a mix of information that is distinctive and information that is common to the different data matrices. To disentangle these sources of variation, we use the rotational freedom of PCA to rotate the metabolite loadings to a partially specified target. We tried all possible types of targets for five components (21 possible patterns of common and distinctive components). Figure 8 displays the deviation from the target against the number of distinctive components. The least deviating solution is the one with two distinctive components for the GC data, two distinctive for the LC data, and one common component.

The proportion of VAF per data block by the five simultaneous components before and after the rotation is shown in Table 4 for each data block. Clearly the rotation reveals the common and distinctive components. Note that rotation does not change the total proportion of VAF by SCA. In Table 4, also the results for the adapted GSVD are shown. These are almost identical to the results obtained with DISCO-SCA, which is confirmed by high average Tucker congruence values (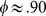) both for the common and block-specific structures. In the remainder of this section, we discuss the interpretation of the DISCO-SCA results.

**Table 4:** Proportion of variance accounted for the mass spectrometry data by the simultaneous components before (SCA) and after rotation (DISCO-SCA) and by the adapted GSVD.

	SCA		DISCO-SCA		Adapted GSVD	
	GC	LC	GC	LC	GC	LC
**C1**	0.07	0.31	0.04	0.31	0.03	0.31
**C2**	0.13	0.13	0.06	0.15	0.07	0.15
**C3**	0.11	0.11	0.16	0.05	0.16	0.05
**C4**	0.12	0.05	0.13	0.04	0.15	0.04
**C5**	0.08	0.08	0.11	0.12	0.11	0.12
**Total**	**0.51**	**0.67**	**0.51**	**0.67**	**0.51**	**0.67**

Proportion of VAF by each of the five simultaneous components (lines C1–C5) and their total (‘Total’) for simultaneous components analysis before (SCA) and after rotation (DISCO-SCA) and for adapted GSVD.

**Figure 9:**
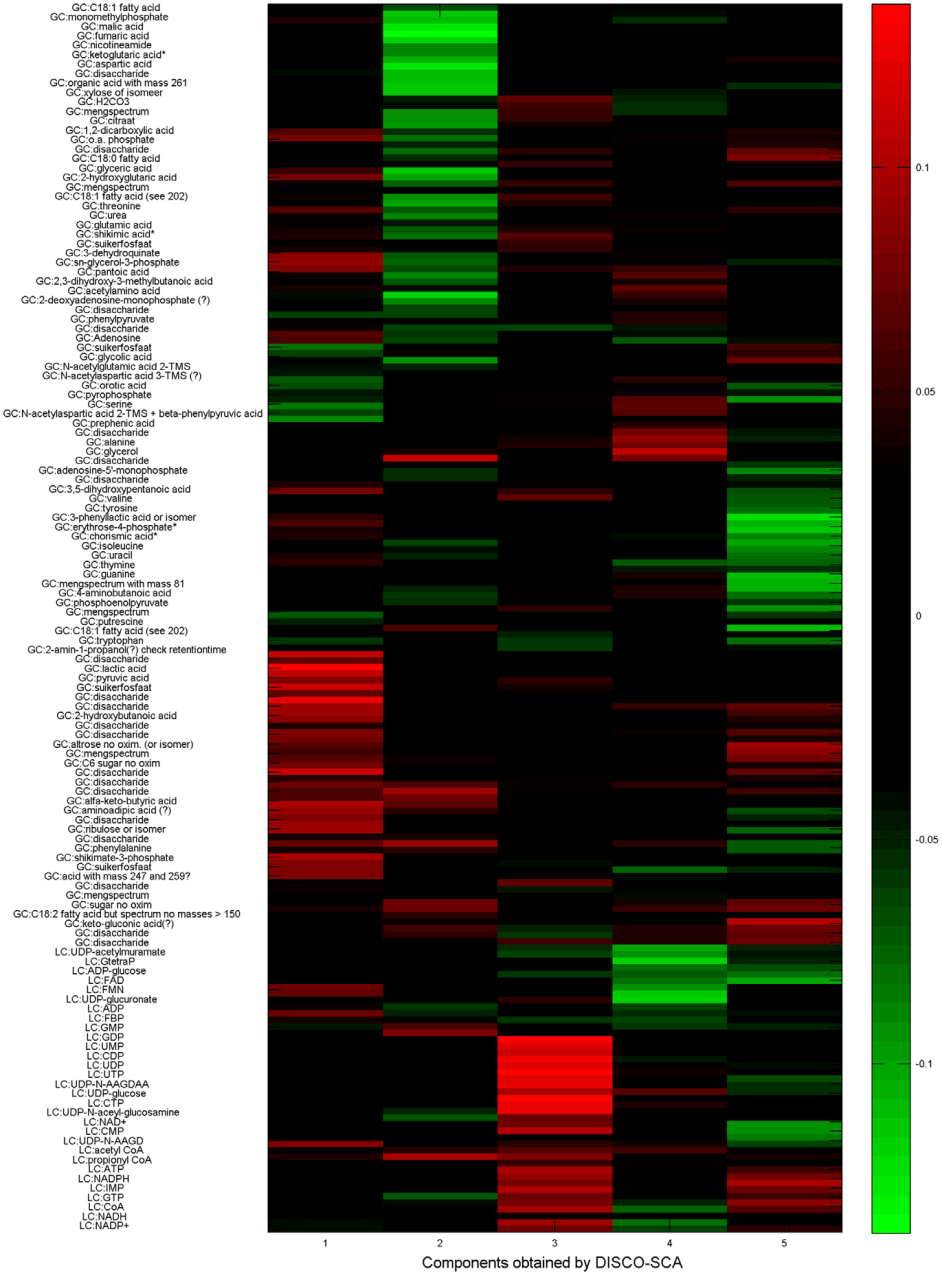
Heatmap of the metabolite loadings on the five common and distinctive components obtained with DISCO-SCA. The heatmap displays the loadings of the metabolites on the five components obtained with DISCO-SCA. The first two components are distinctive for LC, the second two for GC, and the fifth component is common. Upper part: GC data; lower part: LC data.

To interpret the biological processes captured by the simultaneous components, we use the scores of the different fermentation samples on each of the five components (see Table 5) and of the metabolites (see the heatmap of the hierarchically clustered metabolite loadings on the five components in Figure 9). A description of the experimental design underlying these batches can be found in [16], here it is summarized by the first two columns of Table 5 that label the experiments in relation to the reference condition (‘+’ means more than in the reference, ‘−’ less, and ‘oxygen?’ means that not the dissolved oxygen level but the steering speed of the fermenter was controlled) and elapsed fermentation time. The first distinctive component for LC shows mainly an effect of the growth condition with an elevated pH at the early (16 hrs) phase, leading to an abundance of nucleotides important for the energy metabolism in a cell (i.e. AXP, GXP, UXP en CXP). The second LC-specific component shows an effect on the flavin nucleotides FAD and FMN and several other seemingly unrelated nucleotides and CoA esters that are more abundant in conditions with an elevated pH or reduced phosphate level at the mid-logarithmic phase and depleted in the wild type strain. The first specific GC component is associated to the ‘oxygen?’ fermentation condition resulting in a changing, i.e. reduced, dissolved oxygen concentration in the course of the fermentation. Besides a large number of (unidentified) disaccharides, pyruvate and lactate are present in abundance under this condition. This is in agreement with a likely reduced flux through the electron transport chain condition under this environmental condition resulting in the recycling of reducing equivalents via lactate formation. The second specific GC component is associated to succinate catabolism, leading to an increase in concentration of metabolites like fumarate, malate, aspartate, and α-ketoglutarate. This makes biological sense as these metabolites are one or two enzymes removed from succinate in central metabolism. The common component reflects a linear fermentation time effect with very positive scores for the short fermentation times and very negative scores for the long fermentation times. Previously, these data were analyzed by a simultaneous component analysis, followed by a rotation to simple structure [22]. In that analysis, the currently clear presence of two distinctive processes for each separation method and of one common process was not revealed. In addition, the linear effect of fermentation time found here as a mechanism caught by both separation methods, did not show up in this previous analysis.

**Table 5:** Scores of the 28 *E. coli* samples on the DISCO-SCA components.

		LC1	LC2	GC1	GC2	LC-GC
**Reference**	**16**	0.13	−0.02	0.07	0.09	0.29
	**24**	−0.17	−0.04	0.05	0.11	0.10
	**32**	−0.24	0.01	−0.03	0.07	−0.21
	**40**	−0.15	0.10	0.07	0.09	−0.27
	**48**	0.12	0.15	0.04	0.03	−0.22
**pH +**	**16**	−0.63	0.19	0.18	0.17	0.21
	**24**	0.13	0.34	−0.14	0.12	−0.17
	**40**	0.07	0.41	0.10	0.08	−0.20
	**48**	0.09	0.00	0.11	0.13	−0.05
**Oxygen +**	**40**	0.01	−0.09	−0.22	−0.06	0.10
**Oxygen ?**	**16**	0.16	−0.03	0.03	0.20	0.26
	**24**	−0.18	−0.04	−0.26	0.04	0.09
	**40**	−0.05	−0.22	−0.49	−0.10	−0.09
	**64**	−0.05	−0.12	−0.46	−0.12	−0.10
**phosphate +**	**16**	0.19	−0.10	0.07	0.14	0.24
	**24**	0.28	−0.17	−0.02	0.13	0.21
	**40**	−0.07	−0.17	−0.19	−0.09	0.14
	**48**	−0.15	0.11	−0.01	0.03	0.07
**phosphate −**	**16**	−0.02	0.01	−0.03	0.17	0.04
	**24**	0.19	0.28	−0.20	0.08	−0.18
	**40**	0.31	0.13	0.07	0.07	−0.08
**succinate**	**24**	0.02	0.20	0.11	−0.44	0.23
	**40**	0.00	0.16	0.11	−0.53	0.12
	**48**	0.02	0.03	0.14	−0.47	0.03
**Wild type**	**16**	0.13	−0.14	0.21	0.16	0.17
	**24**	−0.21	−0.24	0.18	0.09	−0.02
	**40**	−0.11	−0.32	0.27	−0.09	−0.40
	**48**	0.17	−0.39	0.25	−0.10	−0.31

Scores of the 28 samples on the components. The first two columns describe the experimental design; the next two the scores on the distinctive LC components; the fifth and sixth the scores on the distinctive GC components; and the last the scores on the common component.

## Discussion

The GSVD was proposed as a method to find common and distinctive processes in fused biological data. However, as shown, the GSVD is a full decomposition of the data and does not yield an optimal approximation of the original data by a limited number of components. As an alternative with the property of an optimal approximation by a few components, simultaneous component methods with rotation to common and distinctive components (DISCO-SCA) was proposed. Using simulated data, we showed that DISCO-SCA recovers the underlying common and distinctive processes whereas the GSVD only recovers the distinctive processes which is not the most interesting property since many real-life functional genomics data sets have some degree of overlap or commonness. The use of DISCO-SCA to find common and distinctive processes was illustrated in two applications and compared with the results of a naïve GSVD analysis. First, for a case of comparative genomics with the genome wide expression of human and yeast in synchronized cell cycle experiments, it was shown that the common components found by DISCO-SCA and the adapted GSVD were more enriched for terms related to cell cycle than GSVD. Furthermore, as shown, the performance of the GSVD deteriorates the more experiments or samples are considered. Second, for metabolomics data obtained for the same samples of *E. coli* grown under different environmental conditions but targeted with two different metabolomics platforms (GC-MS and LC-MS): the analysis revealed processes specific for LC (energy metabolism, flavin cofactor related metabolism), specific for GC (recycling of reducing equivalents, succinate catabolism), and common processes (linear time effect). The GSVD analysis accounted in case of the GC data for much less variance (17% compared to 51%).

Interestingly, it appeared that the GSVD can be easily implemented in a way that it yields an optimal approximation of the data. Results of applying the adapted GSVD algorithm to the simulated and empirical data were shown to be highly similar to DISCO-SCA. In fact, there is a close resemblance between the methods. DISCO-SCA is a two-step method, namely SCA and rotation. The GSVD also involves SCA in the first step (in the approach of [15] and in the algorithm of [18]). Furthermore, we showed in section 5 of Information S1 that the adapted GSVD algorithm 1) is equivalent to a simultaneous component analysis also followed by a rotation and 2) imposes the same structure on the data as imposed by a particular simultaneous component analysis model, namely SCA-IND [35].

DISCO-SCA and the GSVD, properly applied, are promising methods to tackle other biological questions such as identifying diverged versus conserved processes in evolutionary biology. In addition, cases with more than two data blocks can be considered. The application of DISCO-SCA to such cases is straightforward. An extension of the GSVD (not adapted!) to find the common components in more than two data blocks has been proposed [36]; an extension that also includes specific components has not yet been developed.

## Supporting Information

Information S1 **Document containing mathematical derivations, simulation studies, and MATLAB code to support the main manuscript.**
(DOC)

Table S1 **Output of GSEA applied to yeast data with the DISCO-SCA components as phenotype.** Only the names for gene sets with FWER p-value<.10 are shown. (none) indicates that no gene set with FWER p-value<.10 was found.
(XLS)

Table S2 **Output of GSEA applied to human data with the DISCO-SCA components as phenotype.** Only the names for gene sets with FWER p-value<.10 are shown. (none) indicates that no gene set with FWER p-value<.10 was found.
(XLS)
