# Supplementary material for: DISCO-SCA and Properly Applied GSVD as Swinging Methods to Find Common and Distinctive Processes
Source: PLoS One. 2012 May 31;7(5):e37840. doi: 10.1371/journal.pone.0037840 (PMC3365060; doi:10.1371/journal.pone.0037840)
Supplement: Information S1 — Document containing mathematical derivations, simulation studies, and MATLAB code to support the main manuscript. (DOC) [file pone.0037840.s001.doc]

SUPPLEMENTARY MATERIAL

The supplementary material is organized in two main parts: one discussing the generalized singular value decomposition (GSVD) more in depth and one containing the MATLAB source code for 1) an (adapted) GSVD algorithm based on the rank determining algorithm of Paige and Saunders (1981), and 2) for the rotation of a matrix to a partially specified target.

# In depth discussion of the GSVD

The main purpose of this part of the supplementary material is to clarify issues that were raised in the paper but not sufficiently explained (due to page constraints) and these issues are:

1) to give theoretical support to the observed inferior performance in terms of least squares of the GSVD compared to DISCO-SCA, especially in the presence of common components (see sections 1 & 2 hereafter),

2) to discuss uniqueness problems for errorless data (see section 3),

3) to generalize the simulation results of the accompanying manuscript (see section 4),

4) to clarify how warranted use of the GSVD results in a well-performing least squares method and show the close links between the adapted GSVD algorithm and simultaneous component analysis via DISCO-SCA and SCA-IND (see section 5).

We start by presenting the GSVD decomposition and the GSVD algorithm of Paige and Saunders (1981).

## Decomposition and algorithm (Paige and Saunders, 1981)

### GSVD decomposition

We consider two data matrices that are coupled in the columns: Let these be denoted by **X**_1_ of size *I*_1_×*J* and **X**_2_ of size *I*_2_×*J*. Also, *Q* is the rank of the concatenated data [**X**_1_ ^T^ **X**_2_ ^T^]^T^. The GSVD decomposition (Paige and Saunders, 1981) of these blocks is then given by

**X**_1_=**U**_1_**S**_1_**V**^T^ (1)

**X**_2_=**U**_2_**S**_2_**V**^T^ , (2)

with **U**_1_ (of size *I*_1_× *I*_1_) and **U**_2_ (of size *I*_2_×*I*_2_) orthogonal, **S**_1_ (*I*_1_×*Q*) and **S**_2_ (*I*_2_×*Q*) matrices with zeros everywhere except for the diagonal positions of the square matrix containing the first *Q* rows and columns of **S**_1_ and the last *Q* rows and columns of **S**_2_. For these diagonal positions, it holds that s²_1qq_+s²_2qq_=1 (see Paige and Saunders, 1981, for a proof). **V** (*J*×*Q*) is a matrix of full rank that represents the common structure shared between **X**_1_ and **X**_2_.

### GSVD algorithm (Paige and Saunders, 1981)

*Step 1: SVD of concatenated data*

**X**_c_=[**X**_1_^T^ **X**_2_^T^]^T^=**U**_c_**S**_c_**V**_c_^T^ (3)

with **X**_c_ of size (*I*_1_+*I*_2_)×*J*, **U**_c_ (of size (*I*_1_+*I*_2_) ×Q) and **V**_c_ (of size *J*×*Q*) orthogonal, and **S**_c_ (*Q*×*Q*) a diagonal matrix with the diagonal elements in a non-increasing order.

*Step 2: SVD of block-specific parts of the left singular vectors* ***U****_c_*

(4) (*I*_1_, *I*_2_, and *Q*: notation to indicate size)

*Step 2a: SVD of* ***U****_c1_* -> gives solution of **U**_1_ and **S**_1_ in (1)

**U**_c1_=**U**_1_**S**_1_**W**^T^ (5)

with **U**_1_ (*I*_1_×*I*_1_) and **W** (*Q*×*Q*) orthogonal and **S**_1_ (*I*_1_×*Q*) as specified in (1).

*Step 2b: SVD of* ***U****_c2_*  -> gives solution of **U**_2_ and **S**_2_ in (2)

**U**_c2_=**U**_2_**S**_2_**W**^T^ (6)

with **U**_2_ (*I*_2_×*I*_2_) orthogonal and **S**_2_ (*I*_2_×*Q*) as specified for (2).

For the proof of the equality of **W** in (5) and (6), see Paige and Saunders.

*Step 3: Derivation of* ***V*** in (1) and (2)

**V**=**V**_c_**S**_c_**W** (7)

## GSVD does not yield the least-squares approximation of [X_1_^T^ X_2_^T^]^T^

Because DISCO-SCA is based on a rotation of the rank *R* approximation obtained by SCA (which is equivalent to a SVD of the concatenated data), it is a least-squares method. This means that for a given number of dimensions/components *R*, it has the lowest sum of squared residuals in approximating the concatenated data [**X**_1_^T^ **X**_2_^T^]^T^. A classical measure to assess the approximation of the model to the data is the complement of the sum of squared residuals, named the variation accounted for (VAF). Note that the VAF by DISCO-SCA is, due to its least-squares property, maximal. *Here, we will show that the VAF by the GSVD components can be expected to be less than the VAF by DISCO-SCA*.

## Expressions for the Variation Accounted For by the GSVD

In this section, we will show that for the GSVD, the VAF by a component *r* can be calculated as **w**_r_^T^**S**_c_²**w**_r_ with **w**_r_ the *r*th column of the matrix **W** in (5) and (6) and **S**_c_² the matrix of squared singular values of the concatenated data.

The Variation Accounted For (VAF) is defined as the data sum of squares minus the sum of squared residuals:

$\begin{matrix} VAF=\sum_{i,j,k} x_{ijk}^{2}-\sum_{i,j,k} \left(x_{ijk}-\hat{x}_{ijk}\right)^{2} \\ =\sum_{k} \left(\left\|\mathbf{X}_{k}\right\|^{2}-\left\|\mathbf{X}_{k}-\hat{\mathbf{X}}_{k}\right\|^{2}\right) \end{matrix}$ (8)

First we show that as for principal and simultaneous component analysis, it *also holds for the GSVD that the VAF can be calculated as the sum of squares of the modeled data*, $\sum_{i,j} \hat{x}_{ijk}^{2}=\left\|\hat{\mathbf{X}}_{k}^{2}\right\|$.

PROOF:

Note that

$\begin{matrix} \mathbf{X}_{1}=\hat{\mathbf{X}}_{1}+\left(\mathbf{X}_{1}-\hat{\mathbf{X}}_{1}\right)=\mathbf{U}_{1R}\mathbf{S}_{1R}\mathbf{V}_{R}^{T}+\mathbf{U}_{1\left(J-R\right)}\mathbf{S}_{1\left(J-R\right)}\mathbf{V}_{\left(J-R\right)}^{T} \\ \mathbf{X}_{2}=\hat{\mathbf{X}}_{2}+\left(\mathbf{X}_{2}-\hat{\mathbf{X}}_{2}\right)=\mathbf{U}_{2R}\mathbf{S}_{2R}\mathbf{V}_{R}^{T}+\mathbf{U}_{2\left(J-R\right)}\mathbf{S}_{2\left(J-R\right)}\mathbf{V}_{\left(J-R\right)}^{T} \end{matrix}$, (9)

with the subscript *R* denoting the *R* model components and *J-R* the remaining components. Given that the terms at the right of (9) are orthogonal (such that $\left\|\mathbf{X}_{k}\right\|^{2}=\left\|\hat{\mathbf{X}}_{k}\right\|^{2}+\left\|\mathbf{X}_{k}-\hat{\mathbf{X}}_{k}\right\|^{2}$) and that both **U**_1_ and **U**_2_ are orthogonal, equality of the VAF to the sum of squared model data, follows directly. Here we give a full development, starting from equation (3):

$\begin{matrix} \sum_{k} \left(\left\|\mathbf{X}_{k}\right\|^{2}-\left\|\mathbf{X}_{k}-\hat{\mathbf{X}}_{k}\right\|^{2}\right)=\left\|\left[\begin{matrix} \mathbf{X}_{1} \\ \mathbf{X}_{2} \end{matrix}\right]\right\|^{2}-\left\|\left[\begin{matrix} \mathbf{X}_{1}-\hat{\mathbf{X}}_{1} \\ \mathbf{X}_{2}-\hat{\mathbf{X}}_{2} \end{matrix}\right]\right\|^{2} \\ =\text{tr}\left(\left[\begin{matrix} \mathbf{X}_{1} \\ \mathbf{X}_{2} \end{matrix}\right]\right)^{T}\left(\left[\begin{matrix} \mathbf{X}_{1} \\ \mathbf{X}_{2} \end{matrix}\right]\right)-\text{tr}\left(\left[\begin{matrix} \mathbf{X}_{1}-\hat{\mathbf{X}}_{1} \\ \mathbf{X}_{2}-\hat{\mathbf{X}}_{2} \end{matrix}\right]\right)^{T}\left(\left[\begin{matrix} \mathbf{X}_{1}-\hat{\mathbf{X}}_{1} \\ \mathbf{X}_{2}-\hat{\mathbf{X}}_{2} \end{matrix}\right]\right) \\ =\text{tr}\left[\mathbf{X}_{1}^{T}\mathbf{X}_{1}+\mathbf{X}_{2}^{T}\mathbf{X}_{2}-\left(\mathbf{X}_{1}^{T}\mathbf{X}_{1}+\hat{\mathbf{X}}_{1}^{T}\hat{\mathbf{X}}_{1}-2\hat{\mathbf{X}}_{1}^{T}\mathbf{X}_{1}+\mathbf{X}_{2}^{T}\mathbf{X}_{2}+\hat{\mathbf{X}}_{2}^{T}\hat{\mathbf{X}}_{2}-2\hat{\mathbf{X}}_{2}^{T}\mathbf{X}_{2}\right)\right] \\ =\text{tr}\left[2\hat{\mathbf{X}}_{1}^{T}\mathbf{X}_{1}-\hat{\mathbf{X}}_{1}^{T}\hat{\mathbf{X}}_{1}+2\hat{\mathbf{X}}_{2}^{T}\mathbf{X}_{2}-\hat{\mathbf{X}}_{2}^{T}\hat{\mathbf{X}}_{2}\right] \\ =\text{tr}\left[2\left(\mathbf{U}_{\text{1R}}\mathbf{S}_{\text{1R}}\mathbf{V}_{\text{R}}^{\text{T}}\right)^{\text{T}}\left(\mathbf{U}_{\text{1R}}\mathbf{S}_{\text{1R}}\mathbf{V}_{\text{R}}^{\text{T}}\text{+}\mathbf{U}_{1\left(\text{J-R}\right)}\mathbf{S}_{1\left(\text{J-R}\right)}\mathbf{V}_{\left(\text{J-R}\right)}^{\text{T}}\right)\text{-}\hat{\mathbf{X}}_{\mathbf{1}}^{\mathbf{T}}\hat{\mathbf{X}}_{\mathbf{1}}+2\left(\mathbf{U}_{\mathbf{2R}}\mathbf{S}_{\mathbf{2R}}\mathbf{V}_{\mathbf{R}}^{\mathbf{T}}\right)^{T}\left(\mathbf{U}_{\mathbf{2R}}\mathbf{S}_{\mathbf{2R}}\mathbf{V}_{\mathbf{R}}^{\mathbf{T}}+\mathbf{U}_{\mathbf{2}\left(\mathbf{J-R}\right)}\mathbf{S}_{\mathbf{2}\left(\mathbf{J-R}\right)}\mathbf{V}_{\left(\mathbf{J-R}\right)}^{\mathbf{T}}\right)-\hat{\mathbf{X}}_{\mathbf{2}}^{\mathbf{T}}\hat{\mathbf{X}}_{\mathbf{2}}\right] \\ =\text{tr}\left[2\left(\mathbf{U}_{1R}\mathbf{S}_{1R}\mathbf{V}_{R}^{T}\right)^{T}\left(\mathbf{U}_{1R}\mathbf{S}_{1R}\mathbf{V}_{R}^{T}\right)+2\left(\mathbf{U}_{1R}\mathbf{S}_{1R}\mathbf{V}_{R}^{T}\right)^{T}\left(\mathbf{U}_{1\left(J-R\right)}\mathbf{S}_{1\left(J-R\right)}\mathbf{V}_{\left(J-R\right)}^{T}\right)-\hat{\mathbf{X}}_{1}^{T}\hat{\mathbf{X}}_{1}\right] \\ +2\text{tr}\left[\left(\mathbf{U}_{2R}\mathbf{S}_{2R}\mathbf{V}_{R}^{T}\right)^{T}\left(\mathbf{U}_{2R}\mathbf{S}_{2R}\mathbf{V}_{R}^{T}\right)+2\left(\mathbf{U}_{2R}\mathbf{S}_{2R}\mathbf{V}_{R}^{T}\right)^{T}\left(\mathbf{U}_{2\left(J-R\right)}\mathbf{S}_{2\left(J-R\right)}\mathbf{V}_{\left(J-R\right)}^{T}\right)-\hat{\mathbf{X}}_{2}^{T}\hat{\mathbf{X}}_{2}\right] \\ =\text{tr}\left[2\left(\mathbf{U}_{1R}\mathbf{S}_{1R}\mathbf{V}_{R}^{T}\right)^{T}\left(\mathbf{U}_{1R}\mathbf{S}_{1R}\mathbf{V}_{R}^{T}\right)+2\mathbf{V}_{R}\mathbf{S}_{1R}\mathbf{U}_{1R}^{T}\mathbf{U}_{1\left(J-R\right)}\mathbf{S}_{1\left(J-R\right)}\mathbf{V}_{\left(J-R\right)}^{T}-\hat{\mathbf{X}}_{1}^{T}\hat{\mathbf{X}}_{1}\right] \\ +2\text{tr}\left[\left(\mathbf{U}_{2R}\mathbf{S}_{2R}\mathbf{V}_{R}^{T}\right)^{T}\left(\mathbf{U}_{2R}\mathbf{S}_{2R}\mathbf{V}_{R}^{T}\right)+2\mathbf{V}_{R}\mathbf{S}_{2R}\mathbf{U}_{2R}^{T}\mathbf{U}_{2\left(J-R\right)}\mathbf{S}_{2\left(J-R\right)}\mathbf{V}_{\left(J-R\right)}^{T}-\hat{\mathbf{X}}_{2}^{T}\hat{\mathbf{X}}_{2}\right] \\ =\text{tr}\left[2\hat{\mathbf{X}}_{1}^{T}\hat{\mathbf{X}}_{1}+\mathbf{0}-\hat{\mathbf{X}}_{1}^{T}\hat{\mathbf{X}}_{1}+2\hat{\mathbf{X}}_{2}^{T}\hat{\mathbf{X}}_{2}+\mathbf{0}-\hat{\mathbf{X}}_{2}^{T}\hat{\mathbf{X}}_{2}\right] \\ =\text{tr}\left[\hat{\mathbf{X}}_{1}^{T}\hat{\mathbf{X}}_{1}+\hat{\mathbf{X}}_{2}^{T}\hat{\mathbf{X}}_{2}\right] \\ =\sum_{k} \left\|\hat{\mathbf{X}}_{k}\right\|^{2}\text{ (10)} \end{matrix}$ which completes the proof.

Using the property that the VAF equals the sum of squared modeled data, the VAF in **X**_1_ and **X**_2_ (or, in the concatenated data **X**_c_=[**X**_1_^T^ **X**_2_^T^]^T^) by GSVD component *r* can be calculated as follows:

$$\begin{matrix} \left\|\hat{\mathbf{X}}_{1}\right\|^{2}+\left\|\hat{\mathbf{X}}_{2}\right\|^{2}=\text{tr}\left(\mathbf{u}_{1r}s_{1rr}\mathbf{v}_{r}^{T}\mathbf{v}_{r}s_{1rr}\mathbf{u}_{1r}^{T}\right)+\text{tr}\left(\mathbf{u}_{2r}s_{2rr}\mathbf{v}_{r}^{T}\mathbf{v}_{r}s_{2rr}\mathbf{u}_{2r}^{T}\right) \\ =\text{tr}\left(s_{1rr}\mathbf{u}_{1r}^{T}\mathbf{u}_{1r}s_{1rr}\mathbf{v}_{r}^{T}\mathbf{v}_{r}\right)+\text{tr}\left(s_{2rr}\mathbf{u}_{2r}^{T}\mathbf{u}_{2r}s_{2rr}\mathbf{v}_{r}^{T}\mathbf{v}_{r}\right) \\ =\text{tr}\left(s_{1rr}^{2}\mathbf{v}_{r}^{T}\mathbf{v}_{r}\right)+\text{tr}\left(s_{2rr}^{2}\mathbf{v}_{r}^{T}\mathbf{v}_{r}\right) \\ =\left(s_{1rr}^{2}+s_{2rr}^{2}\right)\mathbf{v}_{r}^{T}\mathbf{v}_{r} \\ =\mathbf{v}_{r}^{T}\mathbf{v}_{r}\text{ (11)} \end{matrix}$$

Because s²_1rr_+s²_2rr_=1, the derivation also shows that s²_1rr_ and s²_2rr_ can be interpreted as the proportion of VAF by GSVD component *r* respectively in **X**_1_ and **X**_2_.

From (7) and (11), it follows that the VAF for component *r* equals:

$$\begin{matrix} \mathbf{v}_{r}^{T}\mathbf{v}_{r}=\left(\mathbf{V}_{c}\mathbf{S}_{c}\mathbf{w}_{r}\right)^{T}\left(\mathbf{V}_{c}\mathbf{S}_{c}\mathbf{w}_{r}\right) \\ =\mathbf{w}_{r}^{T}\mathbf{S}_{c}\mathbf{V}_{c}^{T}\mathbf{V}_{c}\mathbf{S}_{c}\mathbf{w}_{r} \\ =\mathbf{w}_{r}^{T}\mathbf{S}_{c}^{2}\mathbf{w}_{r}\text{. (12)} \end{matrix}$$

which links the VAF by GSVD component *r* to the VAF by SCA component *r* (namely *s*_cr_²).

## VAF will be in general less than maximal

Note that the VAF by SCA component *r* equals s_cr_², this is the maximal amount component *r* can account for given the previous *r-1* components. The maximal total amount of VAF by *R* components then equals $\sum_{r=1}^{R} s_{cr}^{2}$. So, in order for the *R* GSVD components to account for the same (maximal) VAF, we need to have $\sum_{r=1}^{R} \mathbf{w}_{r}^{T}\mathbf{S}_{c}^{2}\mathbf{w}_{r}=\sum_{r=1}^{R} s_{cr}^{2}$. In general this is not the case. Exceptions are a block-diagonal matrix **W** and the degenerate case that *s*_c1_²=…=*s*_cR_²=…=*s*_cQ_².

Explanation:

Suppose the GSVD components are ordered according to the singular values of the concatenated data. Then the VAF by the *R* GSVD components associated to the *R* largest singular values is,

$$\begin{matrix} \sum_{r=1}^{R} \mathbf{w}_{r}^{T}\mathbf{S}_{c}^{2}\mathbf{w}_{r}=\sum_{r=1}^{R} \left[\begin{matrix} w_{1r} & ... & w_{Rr} & ... & w_{Qr} \end{matrix}\right]\left[\begin{matrix} s_{c1}^{2} & 0 & 0 & 0 & 0 \\ 0 & ... & 0 & 0 & 0 \\ 0 & 0 & s_{cR}^{2} & 0 & 0 \\ 0 & 0 & 0 & ... & 0 \\ 0 & 0 & 0 & 0 & s_{cQ}^{2} \end{matrix}\right]\left[\begin{matrix} w_{1r} \\ ... \\ w_{Rr} \\ ... \\ w_{Qr} \end{matrix}\right] \\ =\sum_{r=1}^{R} \left(w_{1r}^{2}s_{c1}^{2}+...+w_{Rr}^{2}s_{cR}^{2}+...+w_{Qr}^{2}s_{cQ}^{2}\right) \\ =\left(w_{11}^{2}+...+w_{1R}^{2}\right)s_{c1}^{2}+...+\left(w_{R1}^{2}+...+w_{RR}^{2}\right)s_{cR}^{2}+...+\left(w_{Q1}^{2}+...+w_{QR}^{2}\right)s_{cQ}^{2}\text{ (13)} \end{matrix}$$

and this equals $\sum_{r=1}^{R} s_{cr}^{2}$in the exceptional cases a) that *s*_c1_²=…=*s*_cR_²=…=*s*_cQ_² (note that, due to **WW**^T^=**I,** $\sum_{q=1}^{Q} w_{qr}^{2}=1$) or b) that $\sum_{r=1}^{R} \sum_{q=R+1}^{Q} w_{qr}^{2}s_{cq}^{2}=\sum_{r=1}^{R} \sum_{q=R+1}^{Q} w_{rq}^{2}s_{cr}^{2}$. For a block-diagonal **W** matrix (that has *w*_rq_=0=*w*_qr_ with *r*=1…*R* and *q*=*R*+1…*Q*) the latter condition is fulfilled but in general it will not be the case (note that *s*_c1_²>…>*s*_cR_²>…>*s*_cQ_²≥0 and that 0≤*w_qq_*²≤1).

We just illustrated that the GSVD can be expected to have less than maximal VAF for the concatenated data. Also, we hinted at exceptional cases where the GSVD will have maximal VAF. The case of a block-diagonal **W** matrix is of particular interest because it shows up for data with the following special structure: Let **X**_1P_ of rank *R* represent the part of the data matrix **X**_1_ containing the structural information, let **N**_1_ of rank *Q-R* represent the part of **X**_1_ containing only noise, and let these parts be orthogonal to each other. Here, we will show that such data yield a block-diagonal **W** matrix such that the GSVD will have the same optimal least-squares approximation as DISCO-SCA (this is, the same VAF).

If the noise is uncorrelated with the part containing the structural information in the data, we obtain the following result,

$$\begin{matrix} \mathbf{X}_{1}^{T}\mathbf{X}_{1}=\left(\mathbf{X}_{\text{1P}}\text{+}\mathbf{N}_{1}\right)^{T}\left(\mathbf{X}_{\text{1P}}\text{+}\mathbf{N}_{1}\right) \\ =\mathbf{X}_{1P}^{T}\mathbf{X}_{\text{1P}}+\mathbf{N}_{1}^{T}\mathbf{N}_{1}\text{ (14)} \end{matrix}$$

Also, combining (1) and (7),

$$\begin{matrix} \mathbf{X}_{1}^{T}\mathbf{X}_{1}=\left(\mathbf{U}_{1}\mathbf{S}_{1}\mathbf{W}^{T}\mathbf{V}_{c}\mathbf{S}_{c}\right)^{T}\left(\mathbf{U}_{1}\mathbf{S}_{1}\mathbf{W}^{T}\mathbf{V}_{c}\mathbf{S}_{c}\right) \\ =\mathbf{V}_{c}\mathbf{S}_{c}\mathbf{WS}_{1}\mathbf{U}_{1}^{T}\mathbf{U}_{1}\mathbf{S}_{1}\mathbf{W}^{T}\mathbf{S}_{c}\mathbf{V}_{c}^{T} \\ =\mathbf{V}_{c}\mathbf{S}_{c}\mathbf{WS}_{1}\mathbf{S}_{1}\mathbf{W}^{T}\mathbf{S}_{c}\mathbf{V}_{c}^{T} \\ =\left[\begin{matrix} \mathbf{V}_{cR}\mathbf{S}_{cR} & \mathbf{V}_{c\left(Q-R\right)}\mathbf{S}_{c\left(Q-R\right)} \end{matrix}\right]\left[\begin{matrix} \mathbf{W}_{RR}\mathbf{S}_{1R} & \mathbf{W}_{R\left(Q-R\right)}\mathbf{S}_{1\left(Q-R\right)} \\ \mathbf{W}_{\left(Q-R\right)R}\mathbf{S}_{1R} & \mathbf{W}_{\left(Q-R\right)\left(Q-R\right)}\mathbf{S}_{1\left(Q-R\right)} \end{matrix}\right]\left[\begin{matrix} \mathbf{S}_{1R}\mathbf{W}_{RR}^{T} & \mathbf{S}_{1R}\mathbf{W}_{\left(Q-R\right)R}^{T} \\ \mathbf{S}_{1\left(Q-R\right)}\mathbf{W}_{R\left(Q-R\right)}^{T} & \mathbf{S}_{1\left(Q-R\right)}\mathbf{W}_{\left(Q-R\right)\left(Q-R\right)}^{T} \end{matrix}\right]\left[\begin{matrix} \mathbf{S}_{cR}\mathbf{V}_{cR}^{T} \\ \mathbf{S}_{c\left(Q-R\right)}\mathbf{V}_{c\left(Q-R\right)}^{T} \end{matrix}\right] \\ =\left[\begin{matrix} \mathbf{V}_{cR}\mathbf{S}_{cR}\mathbf{W}_{RR}\mathbf{S}_{1R}+\mathbf{V}_{c\left(Q-R\right)}\mathbf{S}_{c\left(Q-R\right)}\mathbf{W}_{\left(Q-R\right)R}\mathbf{S}_{1R} & \mathbf{V}_{cR}\mathbf{S}_{cR}\mathbf{W}_{R\left(Q-R\right)}\mathbf{S}_{1\left(Q-R\right)}+\mathbf{V}_{c\left(Q-R\right)}\mathbf{S}_{c\left(Q-R\right)}\mathbf{W}_{\left(Q-R\right)\left(Q-R\right)}\mathbf{S}_{1\left(Q-R\right)} \end{matrix}\right] \\ \left[\begin{matrix} \mathbf{S}_{1R}\mathbf{W}_{RR}^{T}\mathbf{S}_{cR}\mathbf{V}_{cR}^{T}+\mathbf{S}_{1R}\mathbf{W}_{\left(Q-R\right)R}^{T}\mathbf{S}_{c\left(Q-R\right)}\mathbf{V}_{c\left(Q-R\right)}^{T} \\ \mathbf{S}_{1\left(Q-R\right)}\mathbf{W}_{R\left(Q-R\right)}^{T}\mathbf{S}_{cR}\mathbf{V}_{cR}^{T}+\mathbf{S}_{1\left(Q-R\right)}\mathbf{W}_{\left(Q-R\right)\left(Q-R\right)}^{T}\mathbf{S}_{c\left(Q-R\right)}\mathbf{V}_{c\left(Q-R\right)}^{T} \end{matrix}\right] \\ \text{=}\left(\mathbf{V}_{cR}\mathbf{S}_{cR}\mathbf{W}_{RR}\mathbf{S}_{1R}+\mathbf{V}_{c\left(Q-R\right)}\mathbf{S}_{c\left(Q-R\right)}\mathbf{W}_{\left(Q-R\right)R}\mathbf{S}_{1R}\right)\left(\mathbf{S}_{1R}\mathbf{W}_{RR}^{T}\mathbf{S}_{cR}\mathbf{V}_{cR}^{T}+\mathbf{S}_{1R}\mathbf{W}_{\left(Q-R\right)R}^{T}\mathbf{S}_{c\left(Q-R\right)}\mathbf{V}_{c\left(Q-R\right)}^{T}\right)\text{+} \\ \left(\mathbf{V}_{cR}\mathbf{S}_{cR}\mathbf{W}_{R\left(Q-R\right)}\mathbf{S}_{1\left(Q-R\right)}+\mathbf{V}_{c\left(Q-R\right)}\mathbf{S}_{c\left(Q-R\right)}\mathbf{W}_{\left(Q-R\right)\left(Q-R\right)}\mathbf{S}_{1\left(Q-R\right)}\right)\left(\mathbf{S}_{1\left(Q-R\right)}\mathbf{W}_{R\left(Q-R\right)}^{T}\mathbf{S}_{cR}\mathbf{V}_{cR}^{T}+\mathbf{S}_{1\left(Q-R\right)}\mathbf{W}_{\left(Q-R\right)\left(Q-R\right)}^{T}\mathbf{S}_{c\left(Q-R\right)}\mathbf{V}_{c\left(Q-R\right)}^{T}\right) \\ \text{=}\mathbf{V}_{cR}\mathbf{S}_{cR}\mathbf{W}_{RR}\mathbf{S}_{1R}\mathbf{S}_{1R}\mathbf{W}_{RR}^{T}\mathbf{S}_{cR}\mathbf{V}_{cR}^{T}+\mathbf{V}_{cR}\mathbf{S}_{cR}\mathbf{W}_{RR}\mathbf{S}_{1R}\mathbf{S}_{1R}\mathbf{W}_{\left(Q-R\right)R}^{T}\mathbf{S}_{c\left(Q-R\right)}\mathbf{V}_{c\left(Q-R\right)}^{T}+\mathbf{V}_{c\left(Q-R\right)}\mathbf{S}_{c\left(Q-R\right)}\mathbf{W}_{\left(Q-R\right)R}\mathbf{S}_{1R}\mathbf{S}_{1R}\mathbf{W}_{RR}^{T}\mathbf{S}_{cR}\mathbf{V}_{cR}^{T} \\ +\mathbf{V}_{c\left(Q-R\right)}\mathbf{S}_{c\left(Q-R\right)}\mathbf{W}_{\left(Q-R\right)R}\mathbf{S}_{1R}\mathbf{S}_{1R}\mathbf{W}_{\left(Q-R\right)R}^{T}\mathbf{S}_{c\left(Q-R\right)}\mathbf{V}_{c\left(Q-R\right)}^{T}+\mathbf{V}_{cR}\mathbf{S}_{cR}\mathbf{W}_{R\left(Q-R\right)}\mathbf{S}_{1\left(Q-R\right)}\mathbf{S}_{1\left(Q-R\right)}\mathbf{W}_{R\left(Q-R\right)}^{T}\mathbf{S}_{cR}\mathbf{V}_{cR}^{T}+ \\ \mathbf{V}_{cR}\mathbf{S}_{cR}\mathbf{W}_{R\left(Q-R\right)}\mathbf{S}_{1\left(Q-R\right)}\mathbf{S}_{1\left(Q-R\right)}\mathbf{W}_{\left(Q-R\right)\left(Q-R\right)}^{T}\mathbf{S}_{c\left(Q-R\right)}\mathbf{V}_{c\left(Q-R\right)}^{T}+\mathbf{V}_{c\left(Q-R\right)}\mathbf{S}_{c\left(Q-R\right)}\mathbf{W}_{\left(Q-R\right)\left(Q-R\right)}\mathbf{S}_{1\left(Q-R\right)}\mathbf{S}_{1\left(Q-R\right)}\mathbf{W}_{R\left(Q-R\right)}^{T}\mathbf{S}_{cR}\mathbf{V}_{cR}^{T} \\ +\mathbf{V}_{c\left(Q-R\right)}\mathbf{S}_{c\left(Q-R\right)}\mathbf{W}_{\left(Q-R\right)\left(Q-R\right)}\mathbf{S}_{1\left(Q-R\right)}\mathbf{S}_{1\left(Q-R\right)}\mathbf{W}_{\left(Q-R\right)\left(Q-R\right)}^{T}\mathbf{S}_{c\left(Q-R\right)}\mathbf{V}_{c\left(Q-R\right)}^{T}\text{ (15)} \end{matrix}$$

Applying (14) to this result with the structural part **X**_1P_ coinciding with the first *R* GSVD components (the first term in (15)) and the noise with the last *Q-R* (the last term in (15)), this implies that the sum of all terms in (15) containing a mix of the first *R* and last *Q-R* components should be **0**. Using this result, we have

$$\begin{matrix} \text{tr(}\mathbf{V}_{cR}\mathbf{S}_{cR}\mathbf{W}_{RR}\mathbf{S}_{1R}\mathbf{S}_{1R}\mathbf{W}_{\left(Q-R\right)R}^{T}\mathbf{S}_{c\left(Q-R\right)}\mathbf{V}_{c\left(Q-R\right)}^{T}+\mathbf{V}_{c\left(Q-R\right)}\mathbf{S}_{c\left(Q-R\right)}\mathbf{W}_{\left(Q-R\right)R}\mathbf{S}_{1R}\mathbf{S}_{1R}\mathbf{W}_{RR}^{T}\mathbf{S}_{cR}\mathbf{V}_{cR}^{T}+ \\ \mathbf{V}_{c\left(Q-R\right)}\mathbf{S}_{c\left(Q-R\right)}\mathbf{W}_{\left(Q-R\right)R}\mathbf{S}_{1R}\mathbf{S}_{1R}\mathbf{W}_{\left(Q-R\right)R}^{T}\mathbf{S}_{c\left(Q-R\right)}\mathbf{V}_{c\left(Q-R\right)}^{T}+\mathbf{V}_{cR}\mathbf{S}_{cR}\mathbf{W}_{R\left(Q-R\right)}\mathbf{S}_{1\left(Q-R\right)}\mathbf{S}_{1\left(Q-R\right)}\mathbf{W}_{R\left(Q-R\right)}^{T}\mathbf{S}_{cR}\mathbf{V}_{cR}^{T}+ \\ \mathbf{V}_{cR}\mathbf{S}_{cR}\mathbf{W}_{R\left(Q-R\right)}\mathbf{S}_{1\left(Q-R\right)}\mathbf{S}_{1\left(Q-R\right)}\mathbf{W}_{\left(Q-R\right)\left(Q-R\right)}^{T}\mathbf{S}_{c\left(Q-R\right)}\mathbf{V}_{c\left(Q-R\right)}^{T}+\mathbf{V}_{c\left(Q-R\right)}\mathbf{S}_{c\left(Q-R\right)}\mathbf{W}_{\left(Q-R\right)\left(Q-R\right)}\mathbf{S}_{1\left(Q-R\right)}\mathbf{S}_{1\left(Q-R\right)}\mathbf{W}_{R\left(Q-R\right)}^{T}\mathbf{S}_{cR}\mathbf{V}_{cR}^{T})=\text{tr}\left(\mathbf{0}\right) \\ \Leftrightarrow\text{tr}\left(\mathbf{V}_{c\left(Q-R\right)}^{T}\mathbf{V}_{cR}\mathbf{S}_{cR}\mathbf{W}_{RR}\mathbf{S}_{1R}\mathbf{S}_{1R}\mathbf{W}_{\left(Q-R\right)R}^{T}\mathbf{S}_{c\left(Q-R\right)}\right)+\text{tr}\left(\mathbf{V}_{cR}^{T}\mathbf{V}_{c\left(Q-R\right)}\mathbf{S}_{c\left(Q-R\right)}\mathbf{W}_{\left(Q-R\right)R}\mathbf{S}_{1R}\mathbf{S}_{1R}\mathbf{W}_{RR}^{T}\mathbf{S}_{cR}\right)+ \\ \text{tr}\left(\mathbf{V}_{c\left(Q-R\right)}^{T}\mathbf{V}_{c\left(Q-R\right)}\mathbf{S}_{c\left(Q-R\right)}\mathbf{W}_{\left(Q-R\right)R}\mathbf{S}_{1R}\mathbf{S}_{1R}\mathbf{W}_{\left(Q-R\right)R}^{T}\mathbf{S}_{c\left(Q-R\right)}\right)+\text{tr}\left(\mathbf{V}_{cR}^{T}\mathbf{V}_{cR}\mathbf{S}_{cR}\mathbf{W}_{R\left(Q-R\right)}\mathbf{S}_{1\left(Q-R\right)}\mathbf{S}_{1\left(Q-R\right)}\mathbf{W}_{R\left(Q-R\right)}^{T}\mathbf{S}_{cR}\right)+ \\ \text{tr}\left(\mathbf{V}_{c\left(Q-R\right)}^{T}\mathbf{V}_{cR}\mathbf{S}_{cR}\mathbf{W}_{R\left(Q-R\right)}\mathbf{S}_{1\left(Q-R\right)}\mathbf{S}_{1\left(Q-R\right)}\mathbf{W}_{\left(Q-R\right)\left(Q-R\right)}^{T}\mathbf{S}_{c\left(Q-R\right)}\right)+\text{tr}\left(\mathbf{V}_{cR}^{T}\mathbf{V}_{c\left(Q-R\right)}\mathbf{S}_{c\left(Q-R\right)}\mathbf{W}_{\left(Q-R\right)\left(Q-R\right)}\mathbf{S}_{1\left(Q-R\right)}\mathbf{S}_{1\left(Q-R\right)}\mathbf{W}_{R\left(Q-R\right)}^{T}\mathbf{S}_{cR}\right)=0 \\ \Leftrightarrow\text{tr}\left(\mathbf{S}_{c\left(Q-R\right)}\mathbf{W}_{\left(Q-R\right)R}\mathbf{S}_{1R}\mathbf{S}_{1R}\mathbf{W}_{\left(Q-R\right)R}^{T}\mathbf{S}_{c\left(Q-R\right)}\right)+\text{tr}\left(\mathbf{S}_{cR}\mathbf{W}_{R\left(Q-R\right)}\mathbf{S}_{1\left(Q-R\right)}\mathbf{S}_{1\left(Q-R\right)}\mathbf{W}_{R\left(Q-R\right)}^{T}\mathbf{S}_{cR}\right)=0 \\ \Leftrightarrow\text{tr}\left(\mathbf{S}_{c\left(Q-R\right)}^{2}\mathbf{W}_{\left(Q-R\right)R}\mathbf{S}_{1R}^{2}\mathbf{W}_{\left(Q-R\right)R}^{T}\right)+\text{tr}\left(\mathbf{S}_{cR}^{2}\mathbf{W}_{R\left(Q-R\right)}\mathbf{S}_{1\left(Q-R\right)}^{2}\mathbf{W}_{R\left(Q-R\right)}^{T}\right)=0 \end{matrix}$$

Taking into account that both **S**_c_ and **S**_1_ contain non-zero elements on their diagonal, this means that **W**_R(Q-R)_ and **W**_(Q-R)R_ equal zero. In other words, *data that can be decomposed in two orthogonal parts where one parts contains the structural information of the data, yield a block-diagonal* **W** *matrix and thus maximal VAF by the GSVD approximation*. In practice there is always some correlation of the noise with the structural part of the data, especially for omics data (see Jansen *et al.*, 2005 and Smilde *et al.*, 2009) so in empirical applications it can be expected that the VAF by the GSVD will be less than by DISCO-SCA.

## GSVD performs better for distinctive than for common components

*Why does the presence of (correlated) noise in the data affect less the distinctive than the common components?*

As **U**_c_ is an orthogonal matrix resulting from the SVD of the concatenated data (see (3)), and represents the orthogonal matrix of left singular vectors 1) each vector of **U**_c_ has length one: ||**u**_cr_||=1=||**u**_cr_||²=||**u**_c1r_||²+||**u**_c2r_||² for all *r*, 2) for components that are *distinctive* for **X**_1_ (**X**_2_), the corresponding block-specific values in **u**_c2r_ (**u**_c1r_) are approximately zero, 3) for common components it is not the case that one of the block-specific parts has all values close to zero, and 4) components associated to noise also have ||**u**_cr_||=1. For distinctive components, due to the (near-)zero values in either **u**_c1r_ or **u**_c2r_, orthogonality of **u**_c1r_ (**u**_c2r_) to *any* of the **u**_c1(Q-R)_ (**u**_c2(Q-R)_) will be (approximately) reached. This can be understood as follows: a distinctive component yields all (near-)zero values in either **u**_c1r_ or **u**_c2r_, and a zero-vector is orthogonal to any other vector. Let **u**_c1r_ be the part with all zero values; due to the orthogonality of the concatenated vector **u**_cr_, this implies that also **u**_c2r_ is orthogonal to all other **u**_c2r_ vectors. Furthermore, orthogonality of **U**_c1R_ to **U**_c1(Q-R)_ or of **U**_c2R_ to **U**_c2(Q-R)_ yields a block-diagonal **W** matrix. As explained previously, when **W** is block-diagonal the GSVD yields an optimal approximation. The block-diagonality of **W** can be derived as follows: First, applying the orthogonality to **U**_c1_^T^**U**_c1_,

$$\begin{matrix} \mathbf{U}_{c1}^{T}\mathbf{U}_{c1}=\left[\begin{matrix} \mathbf{U}_{c1R}^{T} \\ \mathbf{U}_{c1\left(Q-R\right)}^{T} \end{matrix}\right]\left[\begin{matrix} \mathbf{U}_{c1R} & \mathbf{U}_{c1\left(Q-R\right)} \end{matrix}\right] \\ =\left[\begin{matrix} \mathbf{U}_{c1R}^{T}\mathbf{U}_{c1R} & \mathbf{0} \\ \mathbf{0} & \mathbf{U}_{c1\left(Q-R\right)}^{T}\mathbf{U}_{c1\left(Q-R\right)} \end{matrix}\right]\text{ (16)} \end{matrix}$$

(A similar result can be obtained for **U**_c2_^T^**U**_c2_). Now for every symmetric block diagonal matrix, the eigendecomposition can obtained directly from the eigendecompositions of the blocks. Specifically, let [**A** **0**; **0** **B**] be the block diagonal matrix in (16), and **A**=**KLK**^T^ and **B**=**MNM**^T^ then [**A** **0**; **0** **B**]=[**K** **0**;**0** **M**][**L** **0**;**0** **N**] [**K** **0**;**0** **M**] ^T^ gives the eigendecompoisition of the block diagonal matrix. Second, note that **W** is the matrix of eigenvectors of **U**_c1_^T^**U**_c1_ (and of **U**_c2_^T^**U**_c2_): **U**_c1_^T^**U**_c1_=( **U**_1_**S**_1_**W**^T^)^T^(**U**_1_**S**_1_**W**^T^) **=WS**_1_**²W**^T^.

The block- orthogonality does not hold for common components. Both for common and noise components the **U***_cr_* are of a non-specific structure and both type of components have length one. Because the GSVD decomposition relies on the SVD of the matrices **U**_c1_ and **U**_c2_ (see (5) and(6)), the common and noise components may be mixed up unlike the distinctive components that have a block-orthogonal structure. This explains the difference in performance of the GSVD for common (bad performance) and distinctive (good performance) processes.

## Non-uniqueness problems

In this section we will show that the GSVD decomposition may not be unique for data that are not perturbed by noise. First we discuss the general case and then a particular case that we link to common and distinctive components in sections 4.1 and 4.2.

## In general

The GSVD is also the SVD of **X**_1_**X**_2_^+^ with **X**_2_^+^ the generalized inverse. As the SVD is not unique in case of equal singular values, this implies that the GSVD is not unique in case of equal singular values for the derived matrix **X**_1_**X**_2_^+^ (note that these singular values are called the generalized singular values).

## For data with a common matrix of right singular vectors

In case of data that can be decomposed by a SVD such that the matrix of right singular vectors is equal, non-uniqueness problems can be linked up to properties of the individual data matrices **X**_1_ and **X**_2_ instead of the derived data matrix **X**_1_**X**_2_^+^: As we will show, this is instructive for the particular case of common and distinctive components. **X**_1_ and **X**_2_ can thus be decomposed as follows,

**X**_1_=**U**_1p_**S**_1p_**V**_p_^T^ (17)

**X**_2_=**U**_2p_**S**_2p_**V**_p_^T^ (18)

with **U**_1p_^T^**U**_1p_=**U**_2p_^T^**U**_2p_=**V**_p_^T^**V**_p_=**I**.

We will use the subscript *p* to denote this special type of data and to make the distinction with the GSVD decomposition (see equations (1) and (2)). Now we inspect what happens upon applying Paige & Saunders GSVD algorithm to these data.

Step 1.

To find the singular values and right singular vectors of the concatenated data, the eigendecomposition is used,

$\begin{matrix} \mathbf{X}_{c}^{T}\mathbf{X}_{c}=\left[\begin{matrix} \mathbf{V}_{p}\mathbf{S}_{1p}\mathbf{U}_{1p}^{T} & \mathbf{V}_{p}\mathbf{S}_{2p}\mathbf{U}_{1p}^{T} \end{matrix}\right]\left[\begin{matrix} \mathbf{U}_{1p}\mathbf{S}_{1p}\mathbf{V}_{p}^{T} \\ \mathbf{U}_{2p}\mathbf{S}_{2p}\mathbf{V}_{p}^{T} \end{matrix}\right] \\ =\mathbf{V}_{p}\left(\mathbf{S}_{1p}^{2}+\mathbf{S}_{2p}^{2}\right)\mathbf{V}_{p}^{T}\text{ (19)} \end{matrix}$.

The left singular vectors of these concatenated data can be obtained as follows: from the general decomposition (3), **X**_c_=**U**_c_**S**_c_**V**_c_^T^, it follows that **U**_c_= **X**_c_(**S**_c_**V**_c_^T^)^-1^=**X**_c_**V**_c_**S**_c_^-1^. For the specific case here, **X**_c_=[(**U**_1p_**S**_1p_**V**_p_^T^)^T^ (**U**_2p_**S**_2p_**V**_p_^T^)^T^]^T^ with associated singular values $\left(\mathbf{S}_{1p}^{2}+\mathbf{S}_{2p}^{2}\right)^{1/2}$ and right singular vectors **V**_p_ (as shown by (21)) yielding left singular vectors

$$\begin{matrix} \mathbf{U}_{c}=\left[\begin{matrix} \mathbf{U}_{1p}\mathbf{S}_{1p}\mathbf{V}_{p}^{T} \\ \mathbf{U}_{2p}\mathbf{S}_{2p}\mathbf{V}_{p}^{T} \end{matrix}\right]\mathbf{V}_{p}\left(\mathbf{S}_{1p}^{2}+\mathbf{S}_{2p}^{2}\right)^{-1/2} \\ =\left[\begin{matrix} \mathbf{U}_{1p}\mathbf{S}_{1p}\left(\mathbf{S}_{1p}^{2}+\mathbf{S}_{2p}^{2}\right)^{-1/2} \\ \mathbf{U}_{2p}\mathbf{S}_{2p}\left(\mathbf{S}_{1p}^{2}+\mathbf{S}_{2p}^{2}\right)^{-1/2} \end{matrix}\right]\text{ . (20)} \end{matrix}$$

Step 2.

To find the structures **U**_1_ and **S**_1_ of the GSVD decomposition (see equation (1)), SVD of the block-specific parts of **U**_c_ is used. For the first block this is the SVD of

$\mathbf{U}_{1p}\mathbf{S}_{1p}\left(\mathbf{S}_{1p}^{2}+\mathbf{S}_{2p}^{2}\right)^{-1/2}$.

Note that **S**_1p_(**S**²_1p_+**S**²_2p_)^-1/2^ is a diagonal matrix and that **U**_1p_ is orthogonal. This immediately yields singular values **S**_1_=**S**_1p_(**S**²_1p_+**S**²_2p_)^-1/2^ and **U**_1_=**U**_1p_. The same holds for the second block specific part: **S**_2_=**S**_2p_(**S**²_1p_+**S**²_2p_)^-1/2^. Note that **S**_1_²+**S**_2_²=**I**. The SVD is not unique in case of equal singular values. Equal singular values in **S**_1_ (and thus also in **S**_2_), will occur when the special data here (see (19) and (20)) have proportional pairs (s_1pi_, s_2pi_) of singular values. We show this as follows: Let s_1pi_ and s_2pi_ be the values on position *i*; also, let *c* be a positive scalar and *c*s_1pi_ and *c*s_2pi_ the values on position *j* (*j*≠*i*), then the values on positions *i* and *j* in **S**_1_ are equal (and thus also those in **S**_2_):

$\frac{cs_{1pi}}{\sqrt{c^{2}s_{1pi}^{2}+c^{2}s_{2pi}^{2}}}=\frac{cs_{1pi}}{c\sqrt{s_{1pi}^{2}+s_{2pi}^{2}}}=\frac{s_{1pi}}{\sqrt{s_{1pi}^{2}+s_{2pi}^{2}}}$.

Note that this is the same as saying that the pairs (s_1pi_,s_2pi_) and (s_1pj_,s_2pj_) have the same ratio: s_1pi_/s_2pi_ = *c*s_1pi_ /*c*s_2pi_= s_1pj_/s_2pj_ (or, the generalized singular values are equal).

Remark that although we discussed uniqueness problems for data with the structure in (19) and (20) relying on a particular algorithm, the results for these data hold in general: setting $\mathbf{V}=\mathbf{V}_{p}\left(\mathbf{S}_{1p}^{2}+\mathbf{S}_{2p}^{2}\right)^{-1/2}$, **U**_1_=**U**_1p_, **U**_2_=**U**_2p_, **S_1_=S**_1p_(**S**²_1p_+**S**²_2p_)^-1/2^ and **S**_2_=**S**_2p_(**S**²_1p_+**S**²_2p_)^-1/2^ yields the GSVD decomposition (see (1) and (2)) which is not unique in case of equal generalized singular values.

## Simulations to illustrate the theoretical results

## Data with noise

In the main manuscript, it was stated that the performance of the GSVD deteriorates the more common components are present. There are two potential confounding factors that need to be addressed in order to make this general statement. First, in the paper only three simulated data cases are considered when discussing the performance in terms of the VAF so this might be a coincidence, and second, the true data have proportional singular value pairs which is problematic due to the uniqueness problems discussed in section 1.2.2. Therefore, we show here the results of a simulations study that includes many data cases and with data generated in a way that the singular value pairs are not proportional.

*Generation of the data:*

The simulated data were generated under a model that fits both the GSVD and DISCO-SCA decompositions:

**X**_1_=**U**_R1_**S**_R1_**V**_R_^T^+**E**_1_,

**X**_2_=**U**_R2_**S**_R2_**V**_R_^T^+**E**_2_,

with **U**_1_^T^**U**_1_=**I**, **U**_2_^T^**U**_2_=**I**, **V**^T^**V**=**I**, and with **X**_1_ of size 144×28 and **X**_2_ of size 44×28 and *R*=3. **S**_1_ and **S**_2_ are diagonal matrices defining whether the components are common (equal singular values: s_1rr_=s_2rr_) or distinctive (e.g., to impose a component that is distinctive for **X**_1_, s_1rr_ is set equal to a substantial value and s_2rr_ to zero). The following structure was used for the diagonal matrices:

diag(**S**_1_)=sqrt([ .40 (1-dd)*(.40) .30 ]),

diag(**S**_2_)=sqrt([ (1-dd)*(.40) .45 (1-dd)*(.30)+.01]),

with dd, the degree of distinctiveness, taking the following values: 1, 0.999, 0.99, 0.9, and 0. The values close to one yield a structure with one distinctive component for **X**_2_ and two for **X**_1_, values close to 0 yield a structure with all common components. **E**_1_ and **E**_2_ were generated from a normal distribution with mean zero and variance such that these residual matrices account for 0 percent, 5 percent, or 20 percent of **X**_1_ and **X**_2_ respectively. Both factors (degree of distinctiveness and error level) were crossed, resulting in 15 conditions, and for each condition 100 data sets were generated.


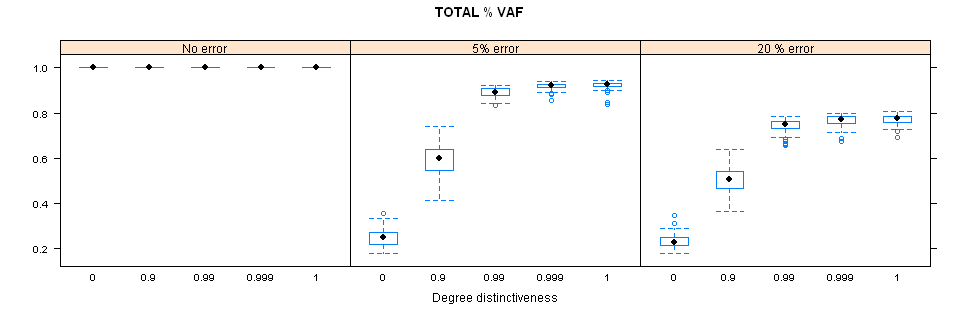


Figure 2: Percentage of VAF accounted for by the GSVD. The different panels refer to different error levels; within each panel, boxplots are shown as a function of different degrees of distinctiveness.

The percentage of VAF is displayed in Figure 2. The trend observed in the article is confirmed: when noise is present and the more common the components become, the worse the approximation of the data. The drop in VAF is quite large: only 20 percent is accounted for when all components are common. Also, already when the degree of distinctiveness is 0.9 (this is 10% of the variation of the component accounted for by the other data block than the one for which the component is distinctive), the performance is considerably affected.


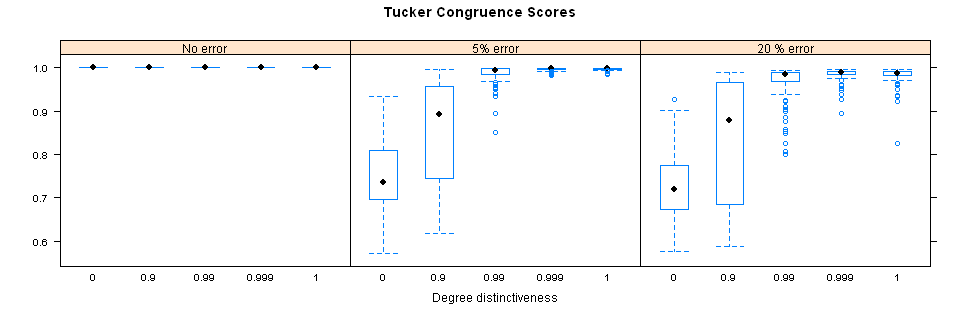


Figure 3: Average Tucker congruence between the columns of the generated and recovered **V** matrix. The different panels refer to different error levels; within each panel, boxplots are shown as a function of different degrees of distinctiveness.


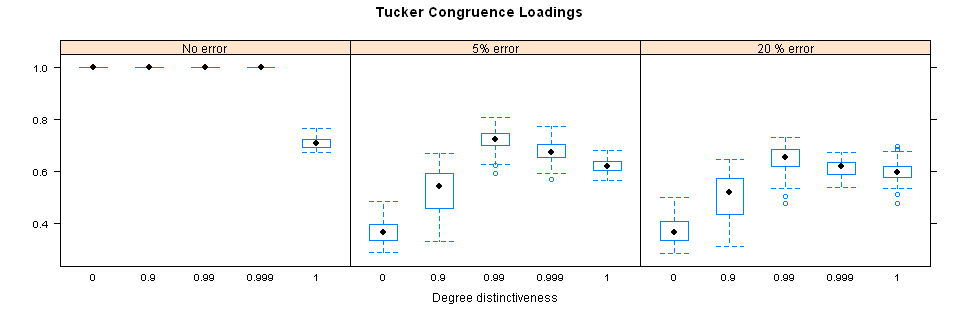


Figure 4: Average Tucker congruence between the columns of the generated and recovered **U**_1_ and **U**_2_ matrix. The different panels refer to different error levels; within each panel, boxplots are shown as a function of different degrees of distinctiveness.

The recovery of the **V** matrix shows the same pattern as the VAF: in presence of error, the performance of the GSVD deteriorates the more common the components are. For the recovery of the **U**_1_ and **U**_2_ matrices, essentially the same trend is observed. However, for perfect distinctive data (degree distinctive = 1 and no error present), the recovery is not perfect. This can be explained by the fact that a distinctive component for **X**_1_ (**X**_2_) plays no role in **X**_2_ (**X**_1_) such that the associated values in **U**_2_ (**U**_1_) are arbitrary. For the same reason, in the panels with error the performance for a degree of distinctiveness equal to 1, the performance is less than for the neighbouring degrees 0.999 and 0.99.

It can be expected that the performance of the GSVD for common components will strongly depend on the number of noise components: The more noise components enter the analysis, the higher the chance that the common directions will correlate with a/some noise directions. We confirmed this in a simulation with 3 common components with clearly non-proportional singular values s_1pi_ and s_2pi_:

**S**_1_=sqrt([.55 .40 .25])

**S**_2_=sqrt([.40 .40 .35])

The factors manipulated are the number of data columns (3, 4, 5, 6, 8, 11, 15, 20, and 30) and the error level (0.1%, 5%, and 20%); see Figure 5. Striking is the observation that the amount of error barely seems to have an influence; *even with only 0.1 percent of error* but a higher rank of the observed than the model data, *the performance deteriorates drastically*.


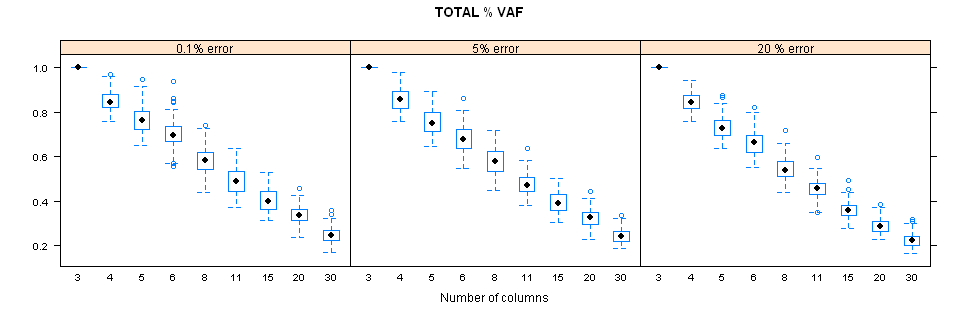


Figure 6. Total percentage of VAF by the three GSVD components with highest VAF for three error levels (the three panels) and a different number of data columns.

## Uniqueness problems

Next we give three randomly generated examples (relying on the model described by equations (16) and (17) with matrices **X**_1_ and **X**_2_ of the same size 144×28 and 44×28 respectively) of uniqueness problems due to proportional pairs of singular values: A first example is general, the second and third example pertain to common and distinctive components.

In Table 1, an example is summarized for data with *R*=5 components and where the fourth and fifth component have proportional singular values. The imposed singular values s_1pi_ and s_2pi_ are summarized by the columns ‘True VAF X1/X2’ and are reported for each of five components (the lines C1-C5). Components four and five have proportional pairs of singular values: s_1p4_/s_2p4_=0.5=s_1p5_/s_2p5_. The values recovered by the GSVD can be found in the columns labeled ‘GSVD VAF X1/X2’: As can be seen, for the fourth and fifth component, the true values are not recovered.

Table 1. Proportion of VAF in each data block by each of five components (the lines C1-C5) and in total (the TOTAL line) as imposed on the data (the columns TRUE VAF) and as reproduced by the GSVD (the columns GSVD VAF).

|  | **TRUE VAF X1** | **TRUE VAF X2** | **GSVD VAF X1** | **GSVD VAF X2** |
| --- | --- | --- | --- | --- |
| **C1** | 0.4000 | 0.3100 | 0.4000 | 0.3100 |
| **C2** | 0.3000 | 0.2900 | 0.3000 | 0.2900 |
| **C3** | 0.2000 | 0.2000 | 0.2000 | 0.2000 |
| **C4** | 0.0800 | 0.1600 | 0.0705 | 0.1409 |
| **C5** | 0.0200 | 0.0400 | 0.0295 | 0.0591 |
| **TOTAL** | **1.0000** | **1.0000** | **1.0000** | **1.0000** |

Perfect common and perfect distinctive data have proportional components. 1) For perfect common data, the proportion of VAF, is the same in each data block yielding s_1pi_/s_2pi_=1 for all *i*. 2) For perfect distinctive data, the VAF in either X_1_ or X_2_ is zero. So, if two components are distinctive for X_1_, this gives two equal s_1pi_/s_2pi_=∞; if two components are distinctive for X_2_, this gives twice s_1pi_/s_2pi_=0. Table 2 shows the results for perfect common data (*R*=4): Only the first component is recovered. Table 3 shows the results for perfect distinctive data (*R*=4) and there only the distinctive components for X1 are recovered (which is probably an algorithmic coincidence; in general it can be expected that also these distinctive components are not recovered).

Table 2. Proportion of VAF in each data block by each of four components (the lines C1-C4) and in total (the TOTAL line) as imposed on the data (the columns TRUE VAF) and as reproduced by the GSVD (the columns GSVD VAF).

|  | **TRUE VAF X1** | **TRUE VAF X2** | **GSVD VAF X1** | **GSVD VAF X2** |
| --- | --- | --- | --- | --- |
| **C1** | 0.5000 | 0.5000 | 0.5000 | 0.5000 |
| **C2** | 0.3000 | 0.3000 | 0.1918 | 0.1918 |
| **C3** | 0.1900 | 0.1900 | 0.1853 | 0.1853 |
| **C4** | 0.0100 | 0.0100 | 0.1230 | 0.1230 |
| **TOTAL** | **1.0000** | **1.0000** | **1.0000** | **1.0000** |

Table 3. Proportion of VAF in each data block by each of four components (the lines C1-C4) and in total (the TOTAL line) as imposed on the data (the columns TRUE VAF) and as reproduced by the GSVD (the columns GSVD VAF).

|  | **TRUE VAF X1** | **TRUE VAF X2** | **GSVD VAF X1** | **GSVD VAF X2** |
| --- | --- | --- | --- | --- |
| **C1** | 0.0000 | 0.7000 | 0.7000 | 0.0000 |
| **C2** | 0.7000 | 0.0000 | 0.0000 | 0.6534 |
| **C3** | 0.0000 | 0.3000 | 0.0000 | 0.3466 |
| **C4** | 0.3000 | 0.0000 | 0.3000 | 0.0000 |
| **TOTAL** | **1.0000** | **1.0000** | **1.0000** | **1.0000** |

*Does the addition of noise to the data solve the uniqueness problem?*

We focus here on distinctive data. Because the addition of noise will disrupt the perfect structure, the distinctive components can be expected to have ratios s_1pi_/s_2pi_ different from zero or infinity. On the one hand, this solves the uniqueness problem but, on the other hand, this may yield arbitrary solutions (in the sense that they are dominated by noise effects) that are far from the true underlying structures. To answer the question whether the addition of noise helps to recover the true underlying structure, we performed a small simulation study.

The data were generated according to the procedure described in the paper but now using randomly generated data to derive the matrices **U**_1_, **U**_2_, and **V**. We consider the situation with only distinctive components (3 for **X**_1_ and 3 for **X**_2_). The noise level was manipulated from zero (no noise) to 20 percent noise. For each level, 100 datasets were generated (this is, generating new **U**_1_, **U**_2_, **V**, **E**_1_, and **E**_2_).
As a measure of performance we used the average Tucker congruence between columns of generated **V** and recovered **V**, and between columns of generated **U**_1_ and **U**_2_ and recovered **U**_1_ and **U**_2_ (in the latter case, the two Tucker congruence values are averaged over **U**_1_ and **U**_2_). Tucker congruence between true matrix **A** and recovered matrix **B** was calculated as follows:

, (21)

after accounting for permutations and reflections.

The results are summarized in Figure 1: the reconstruction of the data is good; however, the recovery of the underlying structures is not good both for the **V** matrix as well as the **U**_1_ and **U**_2_ matrices. Note that for the **V** matrix, Tucker congruence is quite high but given that most error percentages are extremely low, they are not satisfactory. In case of **U**_1_ and **U**_2_, the values are overall low. The most important observation is that the *addition of noise does not improve the recovery*.


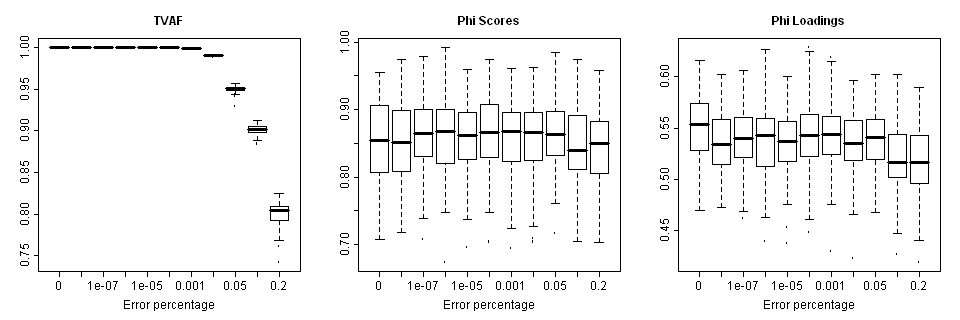


Figure 1: Box plots of total VAF (left panel) and Tucker’s coefficient of congruence (middle panel: **V** matrix; right panel: average for **U**_1_ and **U**_2_ matrices) in function of the error percentage.

## Adapted GSVD algorithm

It can be easily seen that adapting the GSVD algorithm in the following way, yields the least squares approximation of the concatenated data: Namely, limiting **U**_c_ in (4) to the *R* vectors associated to the *R* largest singular values; another approach yielding the same results is to apply the unadapted algorithm of Paige & Saunders or another rank-determining GSVD algorithm to data that are derived from the rank *R* approximation of the concatenated data (see also Friedland, 2005). In fact, the GSVD can then be considered to become a simultaneous component decomposition (step 1 of the algorithm) with additional transformation (steps 2a and 2b). In case the columns refer to the variables, this additional transformation is a rotation and the difference between GSVD and DISCO-SCA then mainly resides in a different rotation criterion. This can be seen as follows.

1. DISCO-SCA starts with SVD of **X**_c_=[**X**_1_^T^ **X**_2_^T^]^T^=**U**_c_**S**_c_**V**_c_^T^ and the block-specific structure **T** is set equal to **U**_cR_=[**U**_cR1_^T^ **U**_cR2_^T^]^T^, the shared structure **P** to **V**_cR_**S**_cR_. Then **T** is rotated by a matrix **B** and **P** is counterrotated by **B**.
2. GSVD starts with SVD of **X**_c_=[**X**_1_^T^ **X**_2_^T^]^T^= **U**_c_**S**_c_**V**_c_^T^.

Then, in step 2a of the GSVD algorithm, **U**_cR1_ is decomposed: **U**_cR1_=**U**_1_**S**_1_**W**^T^ and in step 2b **U**_cR2_=**U**_2_**S**_2_**W**^T^. Note that [(**U**_1_**S**_1_)^T^ (**U**_2_**S**_2_)^T^]^T^= **U**_cR_**W** so the components **U**_1_**S**_1_ and **U**_2_**S**_2_ which can be computed from the **U**_1_, **U**_2_, **S**_1_ and **S**_2_ resulting from steps 2a and 2b can be considered to be a rotation of **U**_cR_ by **W**. The shared structure **V** in the GSVD equals **V**_cR_**S**_cR_**W** which incorporates the counter-rotation of **V**_cR_**S**_cR_.

The results are summarized in Table 4 to clearly show that the only difference between the methods is a difference in rotation: DISCO-SCA rotates to a partially specified target that defines distinctive components by all zero scores (for the other matrix than the one for which the component is distinctive) while GSVD rotates to directions of which the first one accounts for maximal variation in **U**_cR1_ and each subsequent one for the maximal remaining variation. This is for the case the columns refer to the variables (common variables).

Table 4: Comparison of DISCO-SCA and the adapted GSVD algorithm for the case of common variables. **U**_cR_ and **V**_cR_ are the *R* singular vectors corresponding to the *R* largest singular values **S**_cR_ of the concatenated data. **B** and **W** are rotation matrices.

|  | Shared structure | Block-specific structure |
| --- | --- | --- |
| DISCO-SCA | **V**_cR_**S**_cR_**B** | **U**_cR_**B** |
| Adapted GSVD | **V**_cR_**S**_cR_**W** | **U**_cR_**W** |

Interestingly, Timmerman and Kiers (2003) proposed a simultaneous component model (called SCA-IND) for data blocks with a common set of variables that has the following SCA structure:

**X**_1_=**T**_1_**P**^T^ (22)

**X**_2_=**T**_2_**P**^T^, (23)

under the constraints **X**_1_^T^**X**_1_=**PD**_1_²**P**^T^ and **X**_2_^T^**X**_2_=**PD**_2_²**P^T^** with **D**_1_ and **D**_2_ diagonal matrices. Estimation of this model is done in a least-squares framework with **D**_1_²+ **D**_2_²=**I** (in order to identify the solution). For the (adapted) GSVD decomposition, it holds that **X**_1_^T^**X**_1_=**VS**_1_**U**_1_^T^**U**_1_**S**_1_**V**^T^=**VS**_1_²**V**^T^ and, likewise, **X**_2_^T^**X**_2_=**VS**_2_²**V**^T^ with **S**_1_²+**S**_2_²=**I**. This clearly shows that SCA-IND and the (adapted) GSVD impose identical structures on the data blocks **X**_1_ and **X**_2_.

### References

Friedland, S. (2005). A new approach to generalized singular value decomposition. *SIAM Journal on Matrix Analysis and Applications, 27*, 434-444.

Jansen, J.J., Hoefsloot, H.C.J., van der Greef, J., Timmerman, M.E. and Smilde, A.K. (2005). Multilevel Component Analysis of time-resolved metabolic fingerprinting data. Analytica Chimica Acta, 530, 173-183.

Paige,C.C. and Saunders,M.A. (1981). Towards a generalized singular value decomposition. *SIAM J. NUMER. ANAL., 18,* 398-405.

Smilde, A.K. , van der Werf, M.J., Kistemaker, C. and Schaller, J.P. (2009). Characterizing the precision of mass-spectrometry based metabolic profiling platforms, The Analyst, 134, 2281 - 2285.

Timmerman,M.E. and Kiers, H.A.L. (2003). Four simultaneous component models for the analysis of multivariate time series from more than one subject to model intraindividual and interindividual differences. *Psychometrika, 68,* 105-121.

# MATLAB CODE

## GSVD and adapted GSVD algorithm

function [U,V,C,S,X]=gsvdR(A,B,R,full)

%GSVDR GSVD algorithm with possible least squares approach to [A' B']'

%The algorithm is the one proposed by Paige and Saunders (1981) if

%full='yes'; else, it gives the least squares approximation to the

%concatenated data [A' B'] (see Friedland, 2004)

%INPUT: A: first data block

% B: second data block with same number of columns as A

% R: the desired rank of the approximation

% full: 'yes' or 'no' to indicate whether the ordinary GSVD is wanted

% ('yes') or the adapted GSVD with rank R approximation ('no')

%Author: Katrijn Van Deun

[m,n1]=size(A);

[p,n2]=size(B);

if n1==n2

n=n1;

else

fprintf('The data should have the same number of columns \n');

U=[];

V=[];

C=[];

S=[];

X=[];

return;

end;

%Step 1

[P,R0,Q]=svd([A;B]);

nonzerosigma=find(diag(R0)>1e-12);

switch full

case 'yes'

k=size(nonzerosigma,1);

fprintf('Rank of concatenated data is %3.0f \n',k)

case 'no'

k=R;

end;

%Step 2a

P11=P(1:m,1:k);

[U SA W1]=svd(P11);

C=SA;

%Step 2b

P21=P(m+1:m+p,1:k);

[VB SB W]=svd(P21);

kr=min([p k]);

S=zeros(p,k);

S(p:-1:p-kr+1,k:-1:k-kr+1)=SB(1:1:kr,1:1:kr);

V=P21*W1*pinv(S);

Z=zeros(k,n-k);

X=[W1'*R0(1:k,1:k) Z]*Q';

## Partially specified target rotation

function [B Loss]=pstr(T,Target,W,maxiter,convergence)

%PSTR finds a (orthogonal) rotation to a to zero partially specified target

%The following objective function is used (Browne, 1972):

% min_B ||Wo(TB-Target)||²

%with here W a binary matrix of weights, T the matrix to rotate and Target the

%target to reach. There is no problem of reflections due to the restriction

%that the specified numbers are zeros.

%The procedure to minimize the weighted least squares problem implements

%the iterative majorization algorithm proposed by Kiers (1997).

%INPUT T: matrix to rotate

% Target: target to reach

% W: weight matrix (0/1 for resp. unspecified and specified elements of the target)

% maxiter: stop criterion based on maximum number of iterations

% convergence: stop criterion based on difference in loss between current and previous iteration

%OUTPUT B: an orthogonal rotation matrix

% Loss: value of the loss function at termination

%

%Author: Katrijn Van Deun

[n m]=size(T);

L=[];

BMAT=[];

Bcurrent=eye(m);

Lossc=pstrLoss(Bcurrent,T,Target,W);

B1=T'*T;

alpha=max(eig(B1));

iter=1;

stop=0;

while stop==0

Tw=T*Bcurrent-W.*(T*Bcurrent);

A=-2*Tw'*T;

F=A+2*Bcurrent'*B1'-2*alpha*Bcurrent';

[U S V]=svd(-F);

B=V*U';

if iter==maxiter

stop=1;

end;

Loss=pstrLoss(B,T,Target,W);

Diff=Lossc-Loss;

if abs(Diff)<convergence

stop=1;

end;

fprintf('Iteration Nr: %3.0f \t Loss: %5.4f \t Diff: %5.4f \n',iter,Loss,Diff);

iter=iter+1;

Lossc=Loss;

Bcurrent=B;

end;

function Loss=pstrLoss(B,T,Target,W)

%pstrLoss calculates the objective function associated to the pstr function

%

%Author: Katrijn Van Deun

DEV=T*B-Target;

wDEV=W.*DEV;

Loss=sum(sum(wDEV.^2));
